# PQN-59 and GTBP-1 contribute to stress granule formation but are not essential for their assembly in *C. elegans* embryos

**DOI:** 10.1242/jcs.258834

**Published:** 2021-11-15

**Authors:** Simona Abbatemarco, Alexandra Bondaz, Francoise Schwager, Jing Wang, Christopher M. Hammell, Monica Gotta

**Affiliations:** 1 Department of Cell Physiology and Metabolism, Faculty of Medicine, University of Geneva, 1211 Geneva, Switzerland; 2 iGE3 Institute of Genetics and Genomics of Geneva, Geneva, Switzerland; 3 Cold Spring Harbor Laboratory, New York, NY 11724, USA

**Keywords:** *C. elegans*, GTBP-1, PQN-59, UBAP2L, Development, Stress granules

## Abstract

When exposed to stressful conditions, eukaryotic cells respond by inducing the formation of cytoplasmic ribonucleoprotein complexes called stress granules. Here, we use *C. elegans* to study two proteins that are important for stress granule assembly in human cells – PQN-59, the human UBAP2L ortholog, and GTBP-1, the human G3BP1 and G3BP2 ortholog. Both proteins assemble into stress granules in the embryo and in the germline when *C. elegans* is exposed to stressful conditions. Neither of the two proteins is essential for the assembly of stress-induced granules, as shown by the single and combined depletions by RNAi, and neither *pqn-59* nor *gtbp-1* mutant embryos show higher sensitivity to stress than control embryos. We find that *pqn-59* mutants display reduced progeny and a high percentage of embryonic lethality, phenotypes that are not dependent on stress exposure and that are not shared with *gtbp-1* mutants. Our data indicate that, in contrast to human cells, PQN-59 and GTBP-1 are not required for stress granule formation but that PQN-59 is important for *C. elegans* development.

## Abstract (precis)

**Summary:** In contrast to human cells, where the UBAP2L and G3BP1 and G3BP2 proteins are crucial nucleators of stress granules, the *C. elegans* orthologs are not essential for this process in worms.

## INTRODUCTION

Eukaryotic cells are sensitive to changes in internal or environmental parameters, including variations in oxygen supply, salt concentration, pH, temperature and viral infection. Each one of these conditions might be sensed as a stressful stimulus by the cell. In return, cells activate the integrated stress response pathway, which leads to translation inhibition of most mRNAs and to the assembly of stress granules (Kedersha et al., 2013). Stress granules are membraneless organelles formed by the condensation of proteins and RNA molecules into liquid droplets through a mechanism of liquid–liquid phase separation (Hofmann et al., 2021). Different protein entities and RNA molecules are recruited into stress granules, and their composition varies according to the cell type and the triggering stress (Aulas et al., 2017; Markmiller et al., 2018).

Formation of stress-induced granules is a reversible process; hence removal of the stress stimulus results in dissolution of the granules. The current model describing the pathway through which cells assemble stress granules involves disassembly of the polysomes with consequent translation inhibition either via phosphorylation of the translation initiation factor eukaryotic initiation factor 2α (eIF2α) (Kedersha et al., 1999) or via the inhibition of eukaryotic initiation factor 2G (eIF4G) (Mokas et al., 2009). The mRNAs released from the polysomes are then bound to RNA-binding proteins and recruited into the stress granules (Buchan and Parker, 2009; Fay and Anderson, 2018). In mammalian cells, together with the translation initiation factor eIF2, other proteins are important nucleators of stress granules. These include Ras GTPase-activating protein-binding protein 1 and 2 (G3BP1 and G3BP2) and ubiquitin associated protein 2-like (UBAP2L), which are crucial to drive stress granule assembly in many stress conditions (Cirillo et al., 2020; Guillén-Boixet et al., 2020; Huang et al., 2020; Kedersha et al., 2016; Markmiller et al., 2018; Youn et al., 2018), and the protein T-cell-restricted intracellular antigen protein (TIA-1) (Gilks et al., 2004; Kedersha et al., 1999).

Although the exact function of stress granules and their importance for cell survival and organismal development have not yet been established, stress granules may exert a protective role on cells when they are exposed to stress (Protter and Parker, 2016).

Stress granule assembly and function has been mainly studied in unicellular organisms and cells in culture. The nematode *C. elegans* provides an excellent model to study stress granules and to address their role in organismal viability. The proteins involved in stress granule formation in mammalian cells are conserved, and the formation of granules molecularly similar to the mammalian stress granules has been observed in the somatic and germ cells of *C. elegans* (Huelgas-Morales et al., 2016; Jud et al., 2008; Kuo et al., 2020; Lechler et al., 2017; Rousakis et al., 2014).

**Fig. 1.**
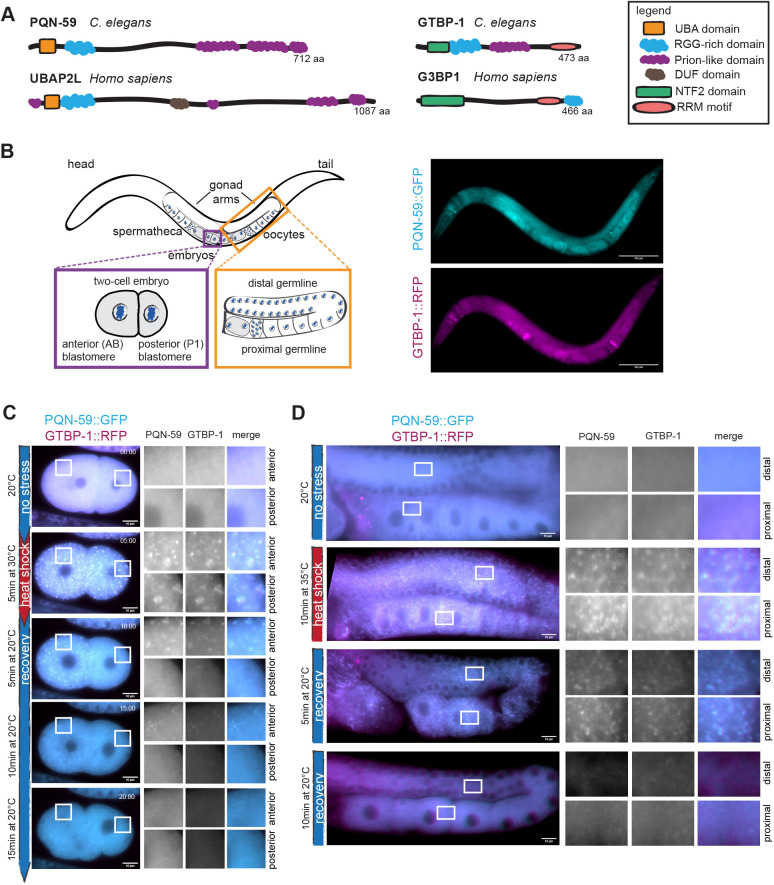
**PQN-59 and GTBP-1 colocalize into heat-stress-induced granules in the embryo and in the germline.** (A) Schematic representation of the protein domains of PQN-59 and of GTBP-1 and their human orthologs, UBAP2L and G3BP1. (B) Left, schematic drawing of an adult *C. elegans* worm, with close ups of a two-cell embryo (purple square) and of the germline (orange square) . Right, images of an adult animal expressing endogenous *pqn-59::GFP;gtbp-1::RFP*. PQN-59 is in cyan, GTBP-1 in magenta. Scale bars: 100 μm. (C) Still frames from time-lapse imaging of *pqn-59::GFP;gtbp-1::RFP* embryos using the CherryTemp temperature-controlled stage. PQN-59 (cyan) and GTBP-1 (magenta) granules were observed in 100% of the observed embryos (*n*=12, *N*=3). Embryos imaging started at 20°C (no stress), the temperature was switched at 30°C (heat shock) and then back to 20°C for the indicated time. The red vertical line on the left shows the time of exposure to heat shock and the blue line the time after stress release (recovery). In all figures, white boxes indicate the ROI shown enlarged on the right. For embryos, ROIs are in the anterior AB cell (top) and in the posterior P1 cell (bottom). (D) Germlines of *pqn-59::GFP;gtbp-1::RFP* worms in control conditions (no stress, 20°C, *n*=5, *N*=3) after heat-stress exposure (10 min at 35°C, *n*=30, *N*=3), after 5 and 10 min recovery (*n*=29 and *n*=20, respectively, *N*=3). PQN-59 (cyan) and GTBP-1 (magenta) form cytoplasmic granules after 10 min of heat exposure at 35°C (red vertical line) and dissolve after 10 min of recovery at 20°C (blue vertical line). In all images of germlines, white boxes in the distal (top) and proximal (bottom) germline show the ROI enlarged on the right. In B–D an epifluorescence microscope was used. Scale bars: 10 μm. In all images, ROIs are enlarged 8× (embryos) and 11.5× (germline). *n* indicates the number of samples and *N* the number of independent experiments.

*C. elegans* contains one ortholog of the mammalian G3BP1 and G3BP2, called GTBP-1 (Sfakianos et al., 2018) and two TIA-1 and TIAR orthologs (Silva-García and Navarro, 2013), named TIAR-1 and TIAR-2. GTBP-1 has been only recently shown to contribute to stress granule formation in *C. elegans* worms (Kuo et al., 2020). TIAR-1 protects germ cells from heat shock (Huelgas-Morales et al., 2016), and TIAR-2 granules inhibit axon regeneration (Andrusiak et al., 2019). The potential *C. elegans* ortholog of UBAP2L is a protein called Prion-like (glutamine/asparagine-rich) domain bearing protein (PQN-59) (Shaye and Greenwald, 2011; Spike et al., 2014). The similarity between PQN-59 and UBAP2L at the sequence level is only 30% (source: BlastP), but PQN-59 and UBAP2L share a very similar domain organization (Fig. 1A). As for GTBP-1, PQN-59 is an abundant protein of the entire *C. elegans* proteome (https://pax-db.org/protein/1033201; Wang et al., 2015; Carlston et al., 2021 preprint), but its role in *C. elegans* has not been characterized.

Here, we show that different stress stimuli trigger the formation of granules containing both PQN-59 and GTBP-1 in *C. elegans* embryos and germlines. In contrast to what has been shown in human cells, we find that neither of the two proteins is essential for stress granule assembly in the embryo, but each contributes to the recruitment of the other to granules. Consistent with this inter-dependence, we show that GTBP-1 and PQN-59 interact in a two-hybrid assay. PQN-59 RNAi depletion or deletion results in embryonic lethality and reduced progeny numbers in normal growth condition, phenotypes that were not observed following the depletion or deletion of GTBP-1. This suggests that PQN-59 plays additional roles in the development of worms.

## RESULTS

### PQN-59 is a component of stress granules

The UBAP2L protein is important for stress granule assembly in many stress conditions and acts upstream of the stress granule components G3BP1 and G3BP2 in this process (Cirillo et al., 2020; Huang et al., 2020; Markmiller et al., 2018; Youn et al., 2018). We set out to investigate whether the *C. elegans* ortholog of UBAP2L, called PQN-59 (Fig. 1A), is also a component of stress granules.

We used a CRISPR/Cas9-generated strain expressing both an endogenous C-terminal fusion of PQN-59 with GFP, and of the ortholog of human G3BP1 and G3BP2, GTBP-1 fused with RFP (Fig. 1A,B; Table S1). Both PQN-59 and GTBP-1 are expressed throughout development, and are widely expressed in adult *C. elegans* animals, including in the germline and the embryo (Fig. 1B).

Observation of untreated *pqn-59::GFP;gtbp-1::RFP* embryos revealed that both proteins are cytoplasmic (in the embryos and in the germline; Fig. 1C,D). When embryos were exposed to heat shock (30°C, 5 min) using a temperature-controlled stage, PQN-59 assembled into granules in both the anterior and posterior blastomeres (Fig. 1C). These granules colocalized with GTBP-1 granules (Fig. 1C). Similar to what is seen for stress granules in human cells (Wheeler et al., 2016), lowering the temperature to 20°C following heat-shock exposure resulted in dissolution of the PQN-59 and GTBP-1 granules after ∼10–15 min of recovery (Fig. 1C).

We investigated whether PQN-59 and GTBP-1 assembled into granules in the *C. elegans* germline following high temperature exposure. We found that in *pqn-59::GFP;gtbp-1::RFP* worms exposed to 35°C for 10 min, PQN-59 assembled into granules in both the distal and proximal germline (Fig. 1D). These granules colocalized with GTBP-1 granules and dissolved after ∼10 min of incubation at 20°C (Fig. 1D), confirming that their formation depends on stress exposure and is reversible. Therefore, heat stress induces the formation of PQN-59- and GTBP-1-containing granules in the *C. elegans* germline.

**Fig. 2.**
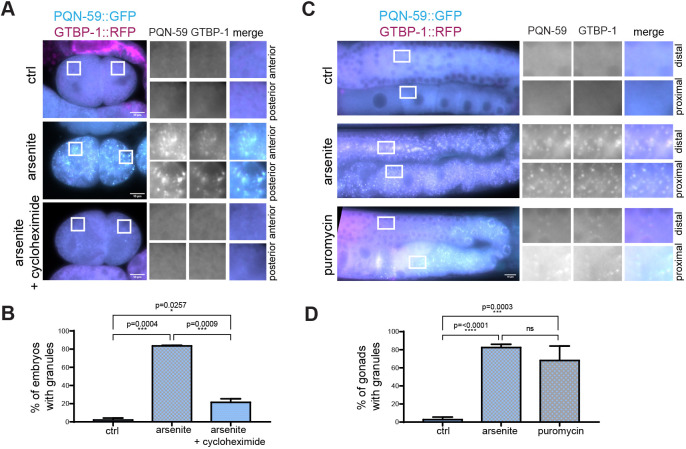
**PQN-59 and GTBP-1 granules form in response to several stresses in the embryo and the germline.** (A) Epifluorescence microscope images of *pqn-59::GFP;gtbp-1::RFP perm-1(RNAi)* embryos (ctrl) and *perm-1(RNAi)* embryos treated with arsenite, or arsenite and cycloheximide as indicated on the left and (B) quantifications of the percentage of embryos with granules (ctrl, *n*=42, arsenite, *n*=143, arsenite and cycloheximide, *n*=87, *N*=2). (C) Epifluorescence microscope images of germlines of *pqn-59::GFP;gtbp-1::RFP* from control and worms treated with arsenite or puromycin, as indicated on the left. (D) Quantifications of the germline showing granules (ctrl, *n*=53, arsenite, *n*=51, puromycin, *n*=58, *N*=4). ROIs are shown enlarged on the right. Error bars indicate s.d. *n* indicates the number of samples and *N* the number of independent experiments.The *P*-value was determined using one-way ANOVA followed by a Tukey's multiple comparisons test. Scale bars: 10 μm.

We then asked whether PQN-59 and GTBP-1 assembled into granules when embryos and worms were exposed to other stresses. Sodium arsenite induces oxidative stress, triggering the formation of stress granules (Rousakis et al., 2014). We treated embryos with *perm-1(RNAi)* to permeabilize the eggshell (Carvalho et al., 2011) and incubated them with 20 mM arsenite for 1 h (Fig. 2A,B). This resulted in appearance of PQN-59 granules that colocalized with GTBP-1 granules in both anterior and posterior cells in 80% of embryos (Fig. 2B). Formation of stress granules relies on polysome disassembly, which is inhibited by the addition of the drug cycloheximide (e.g. Kedersha et al., 2000). Exposure to both arsenite and cycloheximide (250 μg/ml; Lee et al., 2020) inhibited formation of PQN-59 and GTBP-1 granules (Fig. 2A,B), indicating that formation of these granules depends on polysome disassembly.

Germlines from adult worms incubated in a solution containing 20 mM arsenite for 5 h also displayed granules containing both PQN-59 and GTBP-1 (Fig. 2C,D) in the proximal and distal germline. The translation inhibitor puromycin promotes polysome disassembly and stress granule formation (Huelgas-Morales et al., 2016; Kedersha et al., 2000). We asked whether incubation with puromycin induced PQN-59 and GTBP-1 granule formation. As shown in Fig. 2C,D, worms incubated for 4 h in a solution containing 10 mg/ml of puromycin showed the appearance of PQN-59 granules that colocalized with GTBP-1 in the distal and the proximal germline. This suggests that the GTBP-1- and PQN-59-containing granules are stress granules, as they form when polysome disassembly is promoted.

To conclude, the exposure of *C. elegans* embryos and animals to different stress conditions results in the formation of PQN-59 cytoplasmic granules that colocalize with the known stress granule component GTBP-1. Release from heat shock results in granule dissolution and, in embryos, cycloheximide addition inhibits formation of arsenite-induced granules. These data indicate that PQN-59 behaves as a stress granule component.

### The number of GTBP-1 granules is reduced in PQN-59 RNAi depleted embryos

We next asked whether PQN-59 has an important role to form stress granules, as has been shown for UBAP2L (Cirillo et al., 2020; Huang et al., 2020; Markmiller et al., 2018; Youn et al., 2018). To do this we undertook RNAi experiments; for these, silencing of specific genes will be indicated as *gene(RNAi)* or protein name (in capital) RNAi depletion [e.g. *pqn-59(RNAi)* or PQN-59 RNAi depletion, with *ctrl(RNAi)* being *control(RNAi)*].

**Fig. 3.**
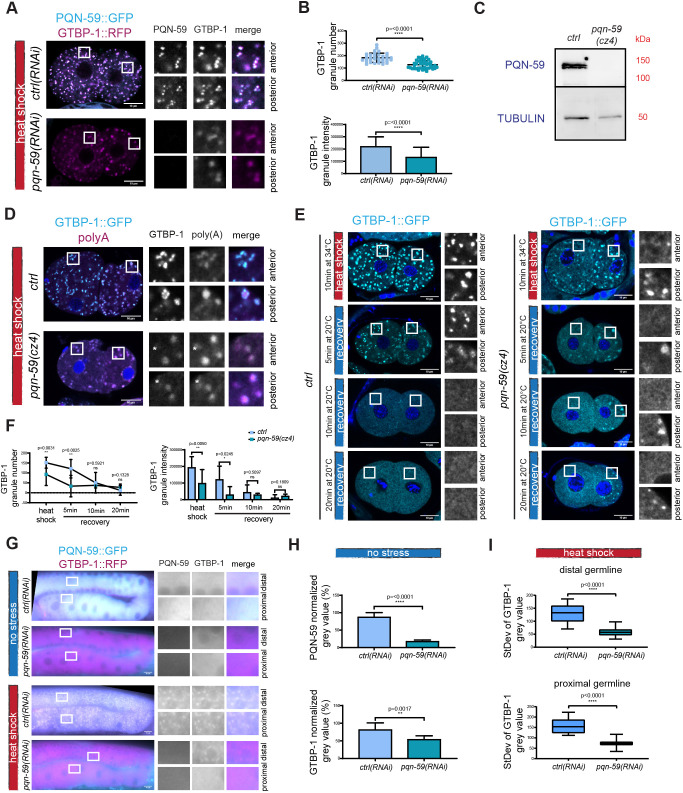
**PQN-59 RNAi depletion results in a reduced number of GTBP-1 stress-induced granules.** (A) Single confocal planes of *pqn-59::GFP;gtbp-1::RFP* fixed two-cell embryos treated with *ctrl* or *pqn-59* RNAi and exposed to heat shock (34°C for 10 min) before fixation. (B) Quantification of the GTBP-1 granule number (top) and the average GTBP-1 granule intensity (bottom) per embryo. *ctrl(RNAi) n*=33; *pqn-59(RNAi) n*=44. *N*=4. (C) Western blot on worm lysates of *gtbp-1::GFP (ctrl)* and *pqn-59(cz4);gtbp-1::GFP* worms using anti-PQN-59 and anti-tubulin (loading control) antibodies. *N*=2. (D) Single confocal planes of *gtbp-1::GFP* and *pqn-59(cz4);gtbp-1::GFP* fixed embryos subjected to FISH for poly(A) RNAs. GTBP-1 GFP signal is in cyan, the poly(A) signal is in magenta and DNA was counterstained with DAPI (blue). Asterisks in the inset indicate poly(A) granules that have very weak or no GTBP-1 signal. *ctrl n*=21, *pqn-59(cz4);gtbp-1::GFP. n*=13. *N*=2. (E) Single confocal planes of *gtbp-1::GFP (ctrl)* and *pqn-59(cz4);gtbp-1::GFP* (*pqn-59(cz4)*) fixed embryos. GTBP-1 GFP signal is in cyan and DNA was counterstained with DAPI (blue). Embryos were fixed at different time points: immediately after heat-shock exposure (10 min at 34°C) and after recovery at 20°C for 5, 10, or 20 min. (F) Quantifications of the dissolution shown in E (left, number of granules at the different time points and, right, of the intensity of the granules. *ctrl*: *n*=12 (HS), *n*=12, *n*=16, *n*=12 (5, 10 and 20 min recovery, respectively); *pqn-59(cz4)*: *n*=9 (HS), *n*= 7, *n*=7, *n*=8 (5, 10 and 20 min recovery, respectively). *N*=4. (G) Epifluorescence microscope images of germlines of *pqn-59::GFP;gtbp-1::RFP* of control or PQN-59 depleted worms, not exposed (top) or exposed (bottom) to heat shock. (H) Quantifications of the cytoplasmic levels of PQN-59 (top) and GTBP-1 (bottom) in the distal germline of non-stressed worms. *ctrl(RNAi) n*=10; *pqn-59(RNAi) n*=11. *N*=2. (I) Quantification of the standard deviation of the GTBP-1 gray value in *control* (*n*=13) and *pqn-59(RNAi)* (*n*=15) distal (top) and proximal (bottom) germlines. *N*=2. Error bars indicate s.d. *P*-values were determined using a two-tailed, unpaired Student's *t*-test. *n* indicates the number of samples and *N* the number of independent experiments. Scale bars: 10 μm. Enlarged ROIs are on the right.

In *ctrl(RNAi)* embryos exposed to 34°C, we observed numerous granules containing PQN-59 and GTBP-1 in both the anterior AB and posterior P1 cells of two-cell embryos (Fig. 3A). When heat shock was applied to *pqn-59(RNAi)* embryos, GTBP-1 assembled into granules in both the anterior and posterior cells (Fig. 3A). Whereas in heat-shocked *ctrl(RNAi)* embryos GTBP-1 granules appeared as spherical and defined speckles, in heat-shocked *pqn-59(RNAi)* embryos, GTBP-1 formed more diffuse granules (Fig. 3A). Quantifications of the GTBP-1 signal revealed that in *pqn-59(RNAi)* embryos, the number and the intensity of GTBP-1 granules were reduced compared to that seen in control embryos (Fig. 3B). The RNAi depletion of PQN-59 was efficient (Fig. 3A; Fig. S1A,B) and, on average, did not result in a change of GTBP-1 levels in embryos, although GTBP-1 levels became more variable (Fig. S1B).

To exclude that a residual pool of PQN-59 after RNAi depletion could account for GTBP-1 granule formation after stress exposure, we inserted a stop codon in the second exon of PQN-59 in a strain expressing GTBP-1::GFP, which resulted in the *pqn-59(cz4);gtbp-1::GFP* strain (Table S1). Expression of PQN-59 was absent in this strain (Fig. 3C). In control embryos exposed to heat shock, we observed GTBP-1 granules (Fig. 3D). These granules contained mRNAs, as shown by fluorescence *in situ* hybridization (FISH) using a poly(A) probe (Fig. 3D), indicating that the PQN-59 and GTBP-1 granules formed after heat shock are ribonucleoprotein complexes, like stress granules. Consistent with the results obtained in the *pqn-59(RNAi)* embryos, in *pqn-59(cz4);gtbp-1::GFP* embryos exposed to heat shock, GTBP-1 assembled into granules, which also contained mRNAs (Fig. 3D). The GTBP-1 granules were, however, less numerous and less intense (Fig. 3D–F), and we could observe poly(A)-positive granules that had very weak or no GTBP-1 signal (marked with asterisks in the magnified images of Fig. 3D).

We noticed that in both *pqn-59(RNAi)* and *pqn-59(cz4)* mutant embryos that were not exposed to heat shock, GTBP-1 formed granules in the posterior P1 blastomere (Fig. S1A,C). These granules colocalized with P body and P granule markers (Fig. S1D,F) and contained mRNAs (Fig. S1G) (Gallo et al., 2008). The average number of P bodies and their intensity was not different in control and *pqn-59(RNAi)* embryos (Fig. S1E) indicating that RNAi depletion of PQN-59 does not affect P body assembly.

Heat-induced GTBP-1 granules dissolved when the temperature was shifted back to 20°C. Images of wild-type embryos fixed after heat shock and after 5, 10 and 20 min of recovery at 20°C, confirmed that GTBP-1 stress-induced granules were still present after 5 min of recovery, and were not detected after 10 min (Fig. 3E,F). In *pqn-59(cz4)* embryos, most GTBP-1 stress-induced granules were already dissolved after 5 min of recovery in the anterior blastomere (Fig. 3E,F). The granules in the posterior blastomere did not dissolve, consistent with the fact that their formation is not dependent on heat-shock exposure (Fig. 3E; Fig. S1A–G).

We then asked whether GTBP-1 granules can form in the germline when PQN-59 is depleted. We found that RNAi depletion of PQN-59 led to a reduction of GTBP-1 levels (Fig. 3G,H). Formation of GTBP-1 granules was not observed in the oocytes (proximal germline) of *pqn-59(RNAi)* worms exposed to heat shock (Fig. 3G,I). However, dim GTBP-1 granules were still observed around the nuclei of the syncytial germline (distal germline, Fig. 3G,I), indicating that, similar to the situation in the embryo, GTBP-1 granules can still form, although not throughout the entire germline, despite the fact that GTBP-1 levels are reduced.

The RGG domain of UBAP2L is crucial to nucleate stress granules in human cells (Huang et al., 2020; Youn et al., 2018). We deleted this domain in the *pqn-59::GFP;gtbp-1::RFP* strain (Fig. S2A; Table S1) and tested whether PQN-59ΔRGG could still form granules after heat shock. As shown in Fig. S2B,C, PQN-59ΔRGG was nucleating granules that colocalized with GTBP-1, similar to the granules formed in the wild-type strain. Quantification of both PQN-59 and GTBP-1 granule number and intensity revealed similar values between the wild-type parental strain and the *pqn-59::ΔRGG::GFP;gtbp-1::RFP* strain (Fig. S2B,C).

Our data show that when PQN-59 is absent, GTBP-1 granules still assemble in the embryo and in the distal germline after heat shock, but not in oocytes. The GTBP-1 granules are reduced in number, appear different from the stress granules assembled in the control strain and dissolve faster. The deletion of the RGG domain of PQN-59 alone is not sufficient to impair granule assembly, indicating that this domain is not essential in this process in *C. elegans* embryos.

### GTBP-1 contributes to the assembly of stress-induced PQN-59 granules

In mammalian cells, the G3BP proteins are crucial to assemble stress granules in many stress conditions (Guillén-Boixet et al., 2020; Kedersha et al., 2016; Sanders et al., 2020; Yang et al., 2020). We therefore investigated whether GTBP-1 was required for the assembly of PQN-59 granules in *C. elegans* after heat shock.

**Fig. 4.**
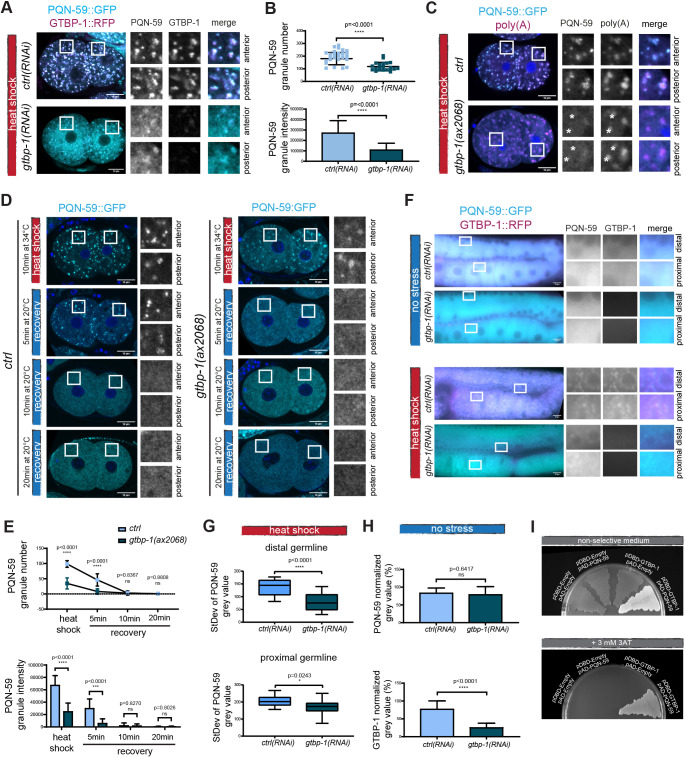
**GTBP-1 RNAi depletion impairs PQN-59 stress-induced granule formation.** (A) Single confocal planes of *pqn-59::GFP;gtbp-1::RFP* fixed two-cell embryos with the indicated genotype and exposed to heat shock (34°C for 10 min) before fixation. (B) Quantification of the average PQN-59 granule number (top) and the average normalized PQN-59 granule intensity (bottom) per embryo. *ctrl(RNAi), n*=40; *gtbp-1(RNAi), n*=22. *N*=4. (C) Single confocal planes of *pqn-59::GFP* and *pqn-59::GFP;gtbp-1(ax2029)* fixed two-cell embryos hybridized with a poly(A) FISH probe. DNA was counterstained with DAPI (blue). Asterisks in the inset indicate poly(A) granules that have very weak or no PQN-59 signal. *ctrl*, *n*=21, *gtbp-1(ax2029), n*=22, *N*=2. (D) Single confocal planes of *pqn-59::GFP* and *pqn-59::GFP;gtbp-1(ax2029)* fixed two-cell embryos. Embryos were fixed at different time points: immediately after heat-shock exposure (red vertical line) and after recovery at 20°C (blue vertical line). (E) Quantifications of PQN-59 granule number (top) and intensity (bottom) at the different time points as shown in D. *ctrl*: *n*=25 (HS), *n*=30, *n*=32, *n*=12 (5, 10 and 20 min recovery, respectively); *gtbp-1(ax2068)*: *n*=27 (HS), *n*=29, *n*=26, *n*=15 (5, 10 and 20 min recovery, respectively). *N*=2. (F) Epifluorescence pictures of control and *gtbp-1(RNAi)* germlines of *pqn-59::GFP;gtbp-1::RFP* worms not exposed (top) or exposed to heat shock (bottom). *ctrl(RNAi) n*=9 (no stress), *n*=15 (HS); *gtbp-1(RNAi) n*=12 (no stress), *n*=16 (HS). *N*=2. (G) Quantification of the standard deviation of the PQN-59 gray value in *ctrl(RNAi)* (*n*=15) and *gtbp-1(RNAi)* (*n*=16) distal (top) and proximal (bottom) germlines. *N*=2. (H) Quantifications of the PQN-59 and GTBP-1 levels in the germlines, *ctrl(RNAi) n*=9, *gtbp-1(RNAi) n*=12. *N*=2. (I) Yeast two-hybrid assay using the PJ69-4a yeast strain transformed with the indicated plasmids. On non-selective plates, all streaks grow. Controls are darker (red) because of lack of interaction and lack of activation of the ADE-2 reporter. The streak of cells containing both PQN-59 and GTBP-1 is white, indicating interaction-dependent activation of the ADE-2 reporter. On selective plates (+3 mM 3AT) yeast growth is observed only for the clone where both PQN-59 and GTBP-1 are expressed, indicating interaction and activation of the HIS-3 reporter. ROIs are enlarged on the right. *n*=3 independent colonies were tested in *N*=2 independent transformations. Error bars indicate s.d. The *P*-value was determined using a two-tailed, unpaired Student's *t*-test. *n* indicates the number of samples and *N* the number of independent experiments. Scale bars: 10 μm. ROIs are enlarged on the right.

We depleted GTBP-1 and imaged the embryos after heat shock and fixation. In *gtbp-1(RNAi)* and *gtbp-1(ax2068)* embryos at 20°C, PQN-59 was diffused in the cytoplasm, as in *ctrl(RNAi)* embryos (Fig. S3A,C). In *gtbp-1(RNAi)* embryos exposed to heat shock, some PQN-59 granules were still observed in both AB and P1 cells (Fig. 4A). A significant decrease in PQN-59 number and intensity could be quantified in GTBP-1-depleted embryos compared to control embryos (Fig. 4B). The dim PQN-59 granules formed in absence of GTBP-1 contained mRNAs, as shown by *in situ* hybridization (Fig. 4C). In addition, we observed poly(A) granules that did not show a clear PQN-59 enrichment (asterisks in the inset of Fig. 4C). In *gtbp-1(ax2068)* embryos that were not exposed to heat shock, the poly(A) signal was detected in P1 cells (Fig. S3C), consistent with a localization in P granules.

The absence of GTBP-1 did not significantly reduce the levels of PQN-59 (Fig. S3A,B), indicating that the impaired stress granule assembly did not depend on a change in protein amount. The stress-induced PQN-59 granules formed in the absence of GTBP-1 also dissolved in a shorter time following stress ceasing (Fig. 4D,E). Whereas in heat-shocked wild-type embryos, granules were still present after 5 min of recovery at 20°C and started to dissolve after 10 min, in *gtbp-1(ax2068)* mutant embryos, the PQN-59 granules already started disappearing after 5 min of recovery at 20°C (Fig. 4D,E), suggesting that the biophysical properties of the granules formed in absence of GTBP-1 are altered compared to control conditions.

RNAi depletion of GTBP-1 also impaired PQN-59 granule assembly in the germline. Following heat exposure, dim PQN-59 granules were still visible around the nuclei in the distal germline. In the proximal germline, undefined PQN-59 aggregates were observed (Fig. 4F,G). Given that PQN-59 levels were not altered following GTBP-1 RNAi depletion (Fig. 4H), this phenotype does not depend on a change in protein amount.

The observation that after PQN-59 RNAi depletion GTBP-1 stress-induced granules are reduced in number and less intense, and that, similarly, after GTBP-1 RNAi depletion PQN-59 granules are reduced in number and less intense suggests an interdependence between these two proteins in their localization to granules. We therefore tested whether PQN-59 and GTBP-1 interact. In agreement with data in other model systems (Baumgartner et al., 2013), and with co-immunoprecipitation analysis from *C. elegans* embryos (Carlston et al., 2021 preprint) PQN-59 interacted with GTBP-1 in two-hybrid assays, as shown by growth on selective medium of yeast colonies expressing GTBP-1 and PQN-59 (Fig. 4I).

We conclude that when GTBP-1 is depleted, stress-exposed embryos contain less numerous and less intense PQN-59 granules. In GTBP-1-depleted germlines, PQN-59 still assembles into granules in the distal and in poorly defined aggregates in the proximal germline.

### TIAR-1 granules assemble in embryos depleted of both GTBP-1 and PQN-59

Since depleting PQN-59 did not abolish formation of GTBP-1 granules and, vice versa, depleting GTBP-1 did not abolish the formation of PQN-59 granules after exposure to stress, we asked whether the absence of both proteins would result in a defect in the formation of stress granules. To address this question, we used the protein TIAR-1 as a marker. In *C. elegans*, TIAR-1 accumulates in stress granules in the germline (Huelgas-Morales et al., 2016) and in the intestine of worms (Kuo et al., 2020) following exposure to different stresses. We used a strain expressing TIAR-1::GFP (Huelgas-Morales et al., 2016) and found that in our heat shock conditions (35°C for 10 min), TIAR-1 also assembled in granules that colocalized with GTBP-1 granules in the distal and proximal germline (Fig. S4A).

**Fig. 5.**
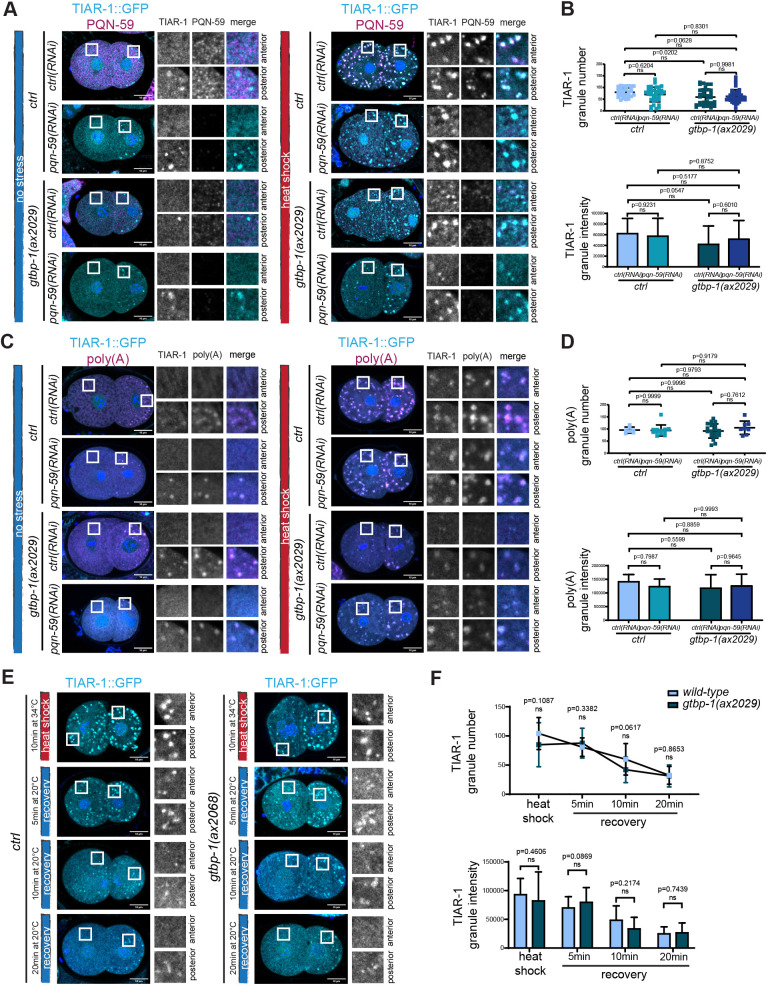
**Formation of TIAR-1 stress-induced granules is not abrogated in *pqn-59(RNAi);gtbp-1(ax2029)* embryos.** (A) Single confocal planes of *tiar-1::GFP* and *tiar-1::GFP;gtbp-1(ax2029)* fixed two-cell embryos treated with the indicated RNAi and immunostained with PQN-59 antibodies (magenta). TIAR-1 GFP signal is in cyan and DNA was counterstained with DAPI (blue). Embryos were kept at 20°C (left) or exposed to heat shock (34°C for 10 min, right) before fixation. For embryos kept at 20°C, *tiar-1::GFP;ctrl(RNAi) n*=16, *tiar-1::GFP*;*pqn-59(RNAi)*, *n*=26, *tiar-1::GFP;gtbp-1(ax2029);ctrl(RNAi) n*=17*, tiar-1::GFP;gtbp-1(ax2029);pqn-59(RNAi) n*=18*.* For embryos exposed to heat shock, *tiar-1::GFP*;*ctrl(RNAi)*, *n*=35, *tiar-1::GFP*;*pqn-59(RNAi) n*=37. In the *tiar-1::GFP;gtbp-1(ax2029), ctrl(RNAi)*, *n*=35, *pqn-59(RNAi) n*=38. *N*=4*.* (B) Quantification of the average TIAR-1 granule number (top) and the average normalized TIAR-1 granule intensity (bottom) per embryo. In the control strain, *ctrl(RNAi)*, *n*=35, *pqn-59(RNAi) n*=37. In the *tiar-1::GFP;gtbp-1(ax2029), ctrl(RNAi)*, *n*=35, *pqn-59(RNAi) n*=38. *N*=3. (C) Single confocal planes of *tiar-1::GFP* and *tiar-1::GFP;gtbp-1(ax2029)* fixed two-cell embryos treated with the indicated RNAi and hybridized with a poly(A) FISH probe. TIAR-1 GFP signal is in cyan, poly(A) in magenta and DNA was counterstained with DAPI (blue). Embryos were kept at 20°C (left) or exposed to heat shock (34°C for 10 min, right) before fixation. For embryos kept at 20°C, *tiar-1::GFP*;*ctrl(RNAi) n*=6, *tiar-1::GFP*;*pqn-59(RNAi)*, *n*=10, *tiar-1::GFP;gtbp-1(ax2029);ctrl(RNAi) n*=12*, tiar-1::GFP;gtbp-1(ax2029);pqn-59(RNAi) n*=8*.* For embryos exposed to heat shock, *tiar-1::GFP*;*ctrl(RNAi) n*=7*, tiar-1::GFP*;*pqn-59(RNAi)*, *n*=12, *tiar-1::GFP;gtbp-1(ax2029);ctrl(RNAi) n*=21*, tiar-1::GFP;gtbp-1(ax2029);pqn-59(RNAi) n*=8. *N*=2. (D) Quantifications of the average number (top) and of the average intensity (bottom) of poly(A) granules. In the control strain, *ctrl(RNAi)*, *n*=7, *pqn-59(RNAi) n*=12. In the *tiar-1::GFP;gtbp-1(ax2029);ctrl(RNAi)*, *n*=21, *pqn-59(RNAi) n*=8. *N*=2. All error bars indicate s.d. The *P*-value was determined using two-way ANOVA test followed by Šídák's multiple comparisons test for the granule number and a Tukey's multiple comparisons test in (B,D) and two-tailed, unpaired Student's t-test (F). *n* indicates the number of samples and *N* the number of independent experiments. Scale bars: 10 μm. Enlarged ROIs are on the right.

In the *C. elegans* embryo, TIAR-1 is localized in the cytoplasm and it accumulates in the nuclei and the P granules of the germ precursor cells (Fig. 5A,C; Huelgas-Morales et al., 2016; Silva-García and Navarro, 2013). Following heat shock of the embryo, TIAR-1 accumulated into stress-induced granules which colocalized with PQN-59 (Fig. 5A). RNAi depletion of PQN-59 did not abolish TIAR-1 granule formation after heat-stress exposure (Fig. 5A). Our quantifications showed that the number and the intensity of TIAR-1 granules was not significantly different compared to the control (Fig. 5B). However, we observed a higher variability in the PQN-59-depleted embryos, consistent with the fact that some embryos appeared to have fewer TIAR-1 granules. In *tiar-1::GFP;gtbp-1(ax2029);ctrl(RNAi)* embryos that were heat shocked, the majority of TIAR-1 granules were detected in the P1 cell (Fig. 5A), but the overall number and intensity of TIAR-1 granules was not significantly different from the wild-type parental strain (Fig. 5B). In this condition, consistent with the result shown in Fig. 4A,B, PQN-59 formed granules in both AB and P1 cells, and these granules colocalized with TIAR-1 granules (Fig. 5A). When PQN-59 was depleted in the *tiar-1::GFP;gtbp-1(ax2029)* embryos, TIAR-1 granules were still observed after heat shock, and their number and intensity was not significantly different from the number and intensity quantified in the control and in the *gtbp-1(ax2029)* mutant (Fig. 5B). The TIAR-1 granules that formed in the *tiar-1::GFP;gtbp-1(ax2029);pqn-59(RNAi)* embryos contained mRNAs as shown by FISH with a poly(A) probe (Fig. 5C). The number and intensity of poly(A) granules was also not significantly different for either the single or the double mutant (Fig. 5D). This suggests that the absence of both PQN-59 and GTBP-1 proteins is not sufficient to abolish the assembly of TIAR-1 stress-induced granules. In embryos that were not exposed to heat shock, the localization and appearance of TIAR-1 was not affected by the RNAi depletion of PQN-59, the mutation of GTBP-1 or both, and the poly(A) probe colocalized with TIAR-1 granules in the P1 cell (Fig. 5A,C). Given that we did not observe a significant difference in TIAR-1 granule number and intensity between the single and double mutant, we measured TIAR-1 granule dissolution in *tiar-1::GFP* and in *tiar-1::GFP;gtbp-1(ax2029)* embryos. We found that the granules dissolved with similar dynamics (Fig. 5E,F), suggesting that the absence of GTBP-1 does not perturb the properties of TIAR-1 granules.

**Fig. 6.**
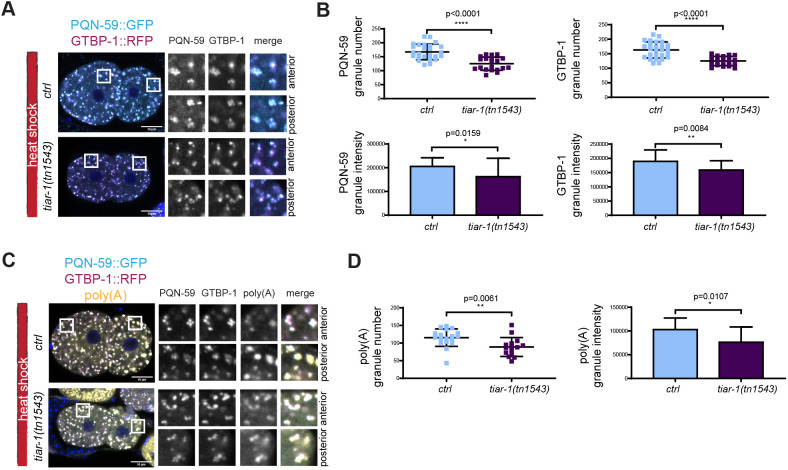
**TIAR-1 contributes to the formation of PQN-59 and GTBP-1 stress-induced granules.** (A) Single confocal planes of *pqn-59::GFP;gtbp-1::RFP* fixed control and *tiar-1(tm1543)* two-cell embryos. PQN-59 is in cyan and GTBP-1 in magenta and (B) quantifications of the number and intensity of GTBP-1 and PQN-5 granules. *ctrl*, *n*=20, *tiar-1(tm1543), n*=26. *N*=2. (C) Single confocal planes of *pqn-59::GFP;gtbp-1::RFP* fixed control and *tiar-1(tm1543)* two-cell embryos hybridized with a poly(A) FISH probe. PQN-59 is in cyan, GTBP-1 in magenta and the poly(A) signal in yellow. (D) Quantifications of the number and intensity of poly(A) granules (as shown in C). *ctrl*, *n*=18, *tiar-1(tm1543), n*=15. *N*=2. Error bars indicate s.d. The *P*-values were determined using two-tailed, unpaired Student's *t*-test. *n* indicates the number of samples and *N* the number of independent experiments. Scale bars: 10 μm. ROIs are enlarged on the right.

We then asked whether formation of PQN-59 and GTBP-1 granules is abolished or reduced when TIAR-1 is mutated. As shown in Fig. 6A,B, the number and intensity of PQN-59 and GTBP-1 granules was reduced in *tiar-1(tn1543)* mutant embryos exposed to heat shock. The number and intensity of poly(A) granules was also reduced in these embryos (Fig. 6C,D). The localization and levels of GTBP-1 and PQN-59 did not change in *tiar-1(tn1543)* embryos not exposed to heat shock (Fig. S4B,C), and the poly(A) signal was found in granules in the posterior P1 cell as in control (Fig. S4D).

TIAR-1 is required to form stress-induced CGH-1 containing granules in the gonad core, but CGH-1-containing granules still form in the oocytes in *tiar-1* mutants (Huelgas-Morales et al., 2016). We found that granules containing both PQN-59 and GTBP-1 still form in germlines from *tiar-1(tn1543)* worms exposed to heat shock (Fig. S4E–G), although the signal from PQN-59 granules appeared reduced (Fig. S4F).

These results indicate that PQN-59 and GTBP-1 are not required for the formation of TIAR-1 granules. TIAR-1 is also not essential for assembly of PQN-59 and GTBP-1 granules but it contributes to it as fewer PQN-59 and GTBP-1 granules assemble in the *tiar-1* mutant.

### PQN-59 is required for embryonic development and maintenance of brood size in a stress-independent manner

As PQN-59 and GTBP-1 contribute to proper granule formation following heat shock, we next investigated whether these two proteins are important for other functions in *C. elegans*, in normal growing conditions and therefore independently of a stress response.

**Fig. 7.**
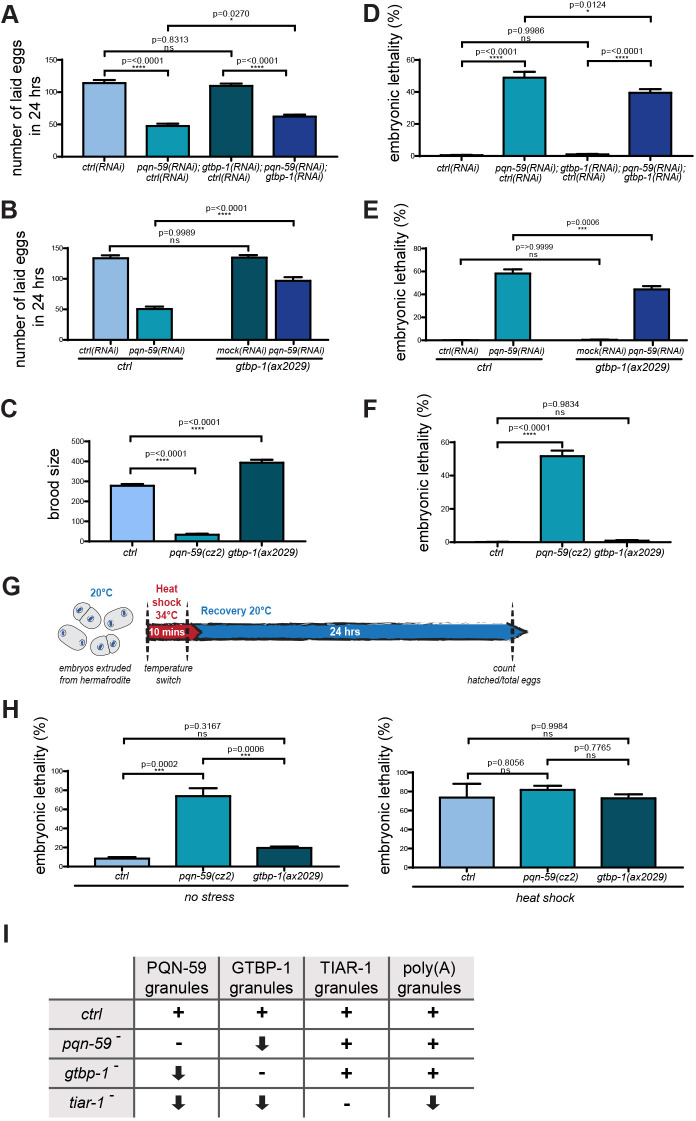
**PQN-59 is important for *C. elegans* embryonic development and brood size.** (A,B) Progeny of *ctrl* after RNAi depletion of the indicated genes in A, and of *ctrl* and *gtbp-1(ax2029)* after RNAi depletion of the indicated genes in B. Values correspond to the average number of eggs laid per single worm in 24 h. (C) Brood size of *ctrl*, *pqn-59(cz2)* and *gtbp-1(ax2029)* strains. (D,E,F) Embryonic lethality of *ctrl* after RNAi depletion of the indicated genes in D, of *ctrl* and *gtbp-1(ax2029)* after RNAi depletion of the indicated genes in E, and of *ctrl*, *pqn-59(cz2)* and *gtbp-1(ax2029)* strains in F. Values correspond to the percentage of non-hatched embryos over the total progeny number (non-hatched embryos and larvae). In A and D, *ctrl(RNAi) n*=3312, *pqn-59;ctrl(RNAi) n*=1430, *gtbp-1(RNAi);ctrl(RNAi) n*=3294, *pqn-59(RNAi);gtbp-1(RNAi) n*=1869. *N*=3. In B and E, *ctrl(RNAi) n*=2007, *pqn-59;ctrl(RNAi) n*=913, *gtbp-1(RNAi);ctrl(RNAi) n*=2022, *pqn-59(RNAi);gtbp-1(RNAi) n*=1742. *N*=2. In C, *ctrl n*=3897, *pqn-59(cz2) n*=605, *gtbp-1(ax2029) n*=7090. *N*=2. In F, *ctrl n*=3301, *pqn-59(cz2) n*=399, *gtbp-1(ax2029) n*=1698. *N*=2. In H, *ctrl n*=103 (no HS), *n*=80 (HS), *pqn-59(cz2) n*=80 (no HS), *n*= 71 (HS), *gtbp-1(ax2029) n*=60 (no HS), *n*=96 (HS). *N*=3. Error bars indicate s.e.m. The *P*-values were determined using one-way ANOVA test followed by a Tukey's multiple comparisons test. (G) Schematic timeline of the experiment to assess embryonic survival after heat shock. In brief, embryos were extruded from gravid hermaphrodites on coverslips, heat shocked and transferred to a NGM plate at 20°C for recovery (see Materials and Methods for more details). (H) Lethality of embryos exposed or not to heat shock was assessed as in D, E and F for *ctrl*, *pqn-59(cz2)* and *gtbp-1(ax2029)* strains counting the non-hatched embryos over the total number of embryos. For *ctrl n*=34, for *pqn-59(cz2) n*=22 and for *gtbp-1(ax2029) n*=18. *N*=2. Error bars indicate s.e.m. The *P*-values were determined using one-way ANOVA test followed by a Tukey's multiple comparisons test. (I) Table summarizing stress-induced granule phenotypes in the different mutants. For each protein, + indicates the wild-type number of granules, − indicates absence of granules, the arrow pointing down indicates a reduction of the number of granules. *n* indicates the number of samples and *N* the number of independent experiments.

We first asked whether the progeny number is affected by the RNAi depletion of PQN-59, GTBP-1 or both. We measured the progeny laid in 24 h and found that RNAi depletion of PQN-59 resulted in a significant reduction of progeny number whereas the RNAi depletion or null mutation of GTBP-1 did not (Fig. 7A,B). Co-depleting both PQN-59 and GTBP-1 or depleting PQN-59 by RNAi in the *gtbp-1* mutant resulted in an increase in brood size compared to that seen with PQN-59 RNAi depletion alone (Fig. 7A,B). We then measured the brood size in *pqn-59* mutant and found that, consistent with the reduced number of eggs laid in 24 h, the brood size was reduced (Fig. 7C). We observed that *gtbp-1* mutant worms, on the opposite, had a significant increase in the brood size compared to control (Fig. 7C).

Depleting by RNAi PQN-59 resulted in ∼50% embryonic lethality (Fig. 7D) a value similar to the PQN-59 mutant (Fig. 7F). These results suggest that PQN-59 has an important function during embryonic development. By contrast, RNAi depletion or mutation of GTBP-1 did not result in significant embryonic lethality (Fig. 7D–F). Depleting GTBP-1 by RNAi did not increase lethality of *pqn-59(RNAi)* embryos compared to the RNAi depletion of PQN-59 alone (Fig. 7D), and rather it weakly rescued (see Discussion). This result was confirmed by the RNAi depletion of PQN-59 in the *gtbp-1* mutant (Fig. 7E).

We then dissected embryos from wild-type and mutant hermaphrodites, exposed them to 34°C for 10 min (Fig. 7G) and analysed how this treatment impacted on their viability. After 24 h of recovery at 20°C, we found that embryonic lethality ranged from ∼70% to ∼80% and we did not detect a significant difference between the wild-type and the mutant embryos (Fig. 7H).

Taken together our results suggest that PQN-59 and GTBP-1 do not help embryos to better resist to exposure to heat. They also indicate that PQN-59 has additional roles in adult life and during development that are independent of GTBP-1 and stress granule formation.

## DISCUSSION

Here, we have studied the function of two conserved proteins, PQN-59, the ortholog of UBAP2L, and GTBP-1, the ortholog of G3BP1 and G3BP2 in assembly of stress granules in worm embryos and in worm germlines. Both PQN-59 and GTBP-1 are cytoplasmic proteins that condense into granules in response to stress exposure (this study; Carlston et al., 2021 preprint; Kuo et al., 2020). While these proteins have been shown to be crucial nucleator of stress granules in human cells (Cirillo et al., 2020; Guillén-Boixet et al., 2020; Huang et al., 2020; Kedersha et al., 2016; Markmiller et al., 2018; Youn et al., 2018), we find that neither the single nor the double RNAi depletion of these proteins result in the abrogation of formation of stress-induced granules in *C. elegans* embryos as detected by TIAR-1::GFP and poly(A) FISH (for a summary of the stress granule phenotypes in the different mutants, see Fig. 7I).

In *Drosophila melanogaster*, Lingerer (the PQN-59 ortholog) and Rasputin (the GTBP-1 ortholog) interact in yeast two-hybrid assays (Baumgartner et al., 2013). In human cells, G3BP-1 and UBAP2L co-immunoprecipitate, and mutations in UBAP2L that abolish the interaction with G3BP-1 are unable to rescue the stress granule assembly defect of UBAP2L RNAi depletion (Huang et al., 2020; Youn et al., 2018). *C. elegans* GTBP-1 has been isolated in pulldowns of PQN-59 from embryos (Carlston et al., 2021 preprint), and we found that PQN-59 and GTBP-1 interact in a yeast two-hybrid assay, supporting the hypothesis that PQN-59 and GTBP-1 are in a complex in *C. elegans*. In contrast with their human orthologs, the interaction of these two proteins or their presence is not essential for the formation of stress-induced granules, as revealed by assessing the behavior of GTBP-1 or PQN-59 and TIAR-1 (Fig. 7I). However, we observed less PQN-59 granules when GTBP-1 was absent and, vice versa, less GTBP-1 granules when PQN-59 was absent (Fig. 7I). This could be either due to a general reduction of the number of stress granules or to the ability of these proteins to be recruited to stress granules when one of them is mutated. The number of TIAR-1 and poly(A) granules did not significantly change in the single or double PQN-59 and GTBP-1 RNAi depletions (Fig. 7I). This indicates that PQN-59 and GTBP-1 are not essential for the assembly of stress granules and suggests that they contribute to, but are not strictly essential for, the recruitment of each other to stress granules. The recruitment in the single mutants may occur via interaction of PQN-59 or GTBP-1 with other stress granule proteins or via binding to RNAs, as we find that the stress-induced granules formed in these mutants do contain mRNAs. The faster dissolution of the PQN-59 granules in the *gtbp-1* mutant and, vice versa, of GTBP-1 granules in the *pqn-59* mutant may suggest that the interaction between these two proteins stabilizes their localization to stress granules but does not affect the properties of stress granules per se. Consistent with this, the dissolution of TIAR-1 granules is not different in control and *gtbp-1* mutant embryos.

Deletion of the RGG domain of UBAP2L results in the abolishment of all interactions with stress granule components and impairs stress granule assembly (Huang et al., 2020). *C. elegans* PQN-59 also contains a RGG domain (Fig. 1A), which is 18% identical and 28% similar to the RGG domain of UBAP2L (aligned using the EMBOSS pairwise alignment). We find that deletion of the PQN-59 RGG domain does not result in defects in the number of stress granules, nor in PQN-59 recruitment to GTBP-1. It is interesting that a deletion of the RGG domain in the P granule component PGL-3 does not abrogate PGL-3 granule formation. PGL-3ΔRGG is, however, unable to recruit other P granule components (Hanazawa et al., 2011). If the RGG domain of PQN-59 were responsible for PQN-59 and/or GTBP-1 recruitment into the stress granules, we would have expected the RGG mutant to show a similar phenotype to the deletion or RNAi depletion of PQN-59. All this suggests that interactions with other stress granule components are maintained in the RGG mutant. It would be therefore interesting to identify the specific domains of PQN-59 involved in its recruitment to granules, interaction with protein partners and/or interaction with RNA molecules.

The single and double RNAi depletion of GTBP-1 and PQN-59 did not reduce the average number of TIAR-1 granules, but granule number was more variable, suggesting that PQN-59 and GTBP-1 do at least partially facilitate TIAR-1 granule formation. This is different from what has been observed in the intestine of *C. elegans* larvae, where GTBP-1 RNAi depletion resulted in a reduction of TIAR-1 granule number but did not fully abolish the formation of stress granules (Kuo et al., 2020). We find that, in the embryo, a mutation in TIAR-1 does not abolish PQN-59 and GTBP-1 stress-induced granule assembly either. However, the number of GTBP-1, PQN-59 and of poly(A) granules was reduced suggesting that TIAR-1 has a more important role in the process of stress granule formation in *C. elegans* embryos. The second *C. elegans* TIA-1 ortholog, TIAR-2, is expressed in the germline (Jud et al., 2008) and has redundant roles with TIAR-1 in regulating brood size and embryonic viability when the temperature is upshifted from 20 to 25°C (Huelgas-Morales et al., 2016). It will be interesting to investigate whether TIAR-1 and TIAR-2 double depletion results in a stronger reduction in GTBP-1, PQN-59 and poly(A) granule formation when embryos are exposed to stress. It will also be interesting to study whether PQN-59 has a more prevalent role in stress granule assembly in other *C. elegans* tissues.

Altogether, our data show that none of these proteins is strictly required for stress-induced granule assembly. So, whereas in cultured human cells G3BPs and UBAP2L are important for the formation of stress granules in many stress conditions (Cirillo et al., 2020; Guillén-Boixet et al., 2020; Huang et al., 2020; Kedersha et al., 2016; Matsuki et al., 2013; Youn et al., 2018), in *C. elegans,* heat-stress-induced granules can form in the embryo in the absence of GTBP-1, PQN-59, in the absence of both and in the absence of TIAR-1. This suggests different possibilities: (1) that an essential nucleator of stress granules has still to be identified in *C. elegans*; (2) that there is strong redundancy; and/or (3) that the presence of disordered proteins is sufficient for the assembly of stress-induced granules in worm embryos. Our work is reminiscent of work in intestinal progenitor cells in *Drosophila* where canonical nucleators are not required for stress granule formation (Buddika et al., 2020). In this system, even a triple mutant of ATX2 (*atx2*), TIAR1 (*rox8*) and G3BP (*rin*) still assembled stress-induced granules as detected by the Fragile X mental retardation protein (FMRP) (Buddika et al., 2020).

Stress granules have been proposed to protect cells from stress. We find that exposure to heat stress kills wild-type, *pqn-59* or *gtbp-1* mutant embryos to the same extent. This is consistent with the result that stress granules still assemble in *pqn-59* and *gtbp-1* mutant embryos and reveals that these two factors are not crucial to protect *C. elegans* embryos from stress. In *tiar-1* mutants, in which we find that GTBP-1, PQN-59 and poly(A) granules are reduced in number, embryonic lethality is increased after heat stress compared to control (Huelgas-Morales et al., 2016), suggesting that having enough stress granules may be important to protect embryos.

RNAi depletion and mutation of PQN-59 result in additional phenotypes, such as slow growth, reduced progeny and embryonic lethality, all in absence of stress. These phenotypes were not observed in *gtbp-1* mutant or depleted animals. A recent paper has shown that the human orthologs, G3BP1 and G3BP2 inhibit mTORC1 signaling by targeting mTORC1 to the lysosome (Prentzell et al., 2021). One possibility is that the phenotypes of *pqn-59* mutant embryos are dependent on GTBP-1. For example, an excess of free GTBP-1 (not in complex with PQN-59) could be deleterious for worms and embryos. In addition, in *pqn-59* mutant embryos not exposed to stress we observed that, in the P1 cell, GTBP-1 localizes to granules that colocalize with P body and P granule markers, suggesting the hypothesis that this aberrant localization is deleterious to embryos. Co-depletion of both PQN-59 and GTBP-1 by RNAi resulted in a weak rescue of the embryonic lethality and the reduced progeny phenotypes of *pqn-59* mutants, indicating that these phenotypes might depend on an excess of free GTBP-1 or on any role that GTBP-1 might play when localized on P bodies/P granules, but only partially. Why GTBP-1 localizes to other granules in absence of stress in *pqn-59* mutants is not clear. One possibility is that, in non-stress conditions, GTBP-1 has a tendency to phase separate stronger than PQN-59. This tendency would normally be inhibited by the presence of PQN-59. An alternative possibility is that the region of GTBP-1 responsible to interact with PQN-59 may also interact, more weakly, with P body or P granule components, an interaction that could be revealed when PQN-59 is absent.

The fact that the rescue of lethality observed in the double RNAi depletion is weak suggests that PQN-59 has additional important functions in embryos and worms that do not depend on GTBP-1. These yet to be identified functions could contribute to the regulation of the response to stress or be completely independent of the role of PQN-59 in stress granule assembly. Additional studies will be required to understand the molecular functions of PQN-59 and its role in maintaining embryonic viability.

## MATERIALS AND METHODS

### Strains

The *C. elegans* strains used in this work are listed in Table S1. Worms were maintained on NGM plates seeded with OP50 bacteria, using standard methods (Brenner, 1974). All the strains were grown at 20°C and incubated at 20°C after dsRNAs injections.

Mutant strains were generated using CRISPR/Cas-9 technology, as described in Arribere et al. (2014). Single-guide RNAs and repair templates, as well as PCR primers used to detect and sequence the mutations, are listed in Tables S2 and S3, respectively. The *pqn-59* mutant strain {generated in the N2 background and in the JH3199 (*gtbp-1(ax2055[gtbp-1::GFP])IV*) background} was generated by introducing a frameshift mutation leading to the appearance of a premature STOP codon. The *pqn-59::ΔRGG* strain was generated excising the RGG-rich region (from amino acid position 122 to 189), not altering the reading frame.

### RNA interference

A list of the genes silenced through RNAi in this study is provided in Table S4.

Clones from the Ahringer feeding library (Ahringer, 2006; Kamath et al., 2003) were used when available. As a control, we used the clone C06A6.2, previously found in the laboratory to not affect early embryonic division and development (injected worms are 100% viable). To produce *pqn-59* dsRNA, a DNA fragment was amplified from genomic DNA using Gateway-compatible oligonucleotide primers (as in Table S4) for Gateway-based-cloning into the pDESTL4440 plasmid (Addgene plasmid #11344). The DNA was subsequently amplified using standard T7 primers. For *tiar-1*, the DNA was amplified from genomic DNA using oligonucleotides with T7 overhangs (see Table S4). For all genes, the dsRNA was produced with the Promega Ribomax RNA production system. dsRNA was injected in L4 or young adult hermaphrodites, which were incubated at 20°C. Germlines or embryos collected from injected hermaphrodites were analyzed 24 h after injection.

### Live imaging of embryos exposed to heat shock

Gravid hermaphrodites were dissected on a coverslip into a drop of egg buffer (118 mM NaCl, 48 mM KCl, 2 mM CaCl_2_, 2 mM MgCl_2_, and 25 mM HEPES pH 7.5) containing a 1:10 volume of polystyrene beads (Polybead^®^ Hollow Microspheres, Polysciences). The temperature controller CherryTemp (Cherry Biotech, Rennes, France) with its accompanying software (Cherry Biotech TC) was used to control the temperature during the live imaging process. The coverslip with dissected hermaphrodites was directly mounted on the chip of the CherryTemp microfluidic temperature control system. The system was mounted on a Leica DM6000 microscope, equipped with epifluorescence and differential interference contrast (DIC) optics, and a DFC 360 FX camera (Leica). Time lapse images were collected every 10 s using a 63×/1.4 numerical aperture (NA) objective and LAS AF software (Leica Biosystems). Imaging was started at 20°C. The temperature was then shifted at 30°C (heat shock) for 5 to 10 min while imaging. For recovery, the temperature was shifted back to 20°C for 15–20 min (Fig. 1C).

### Embryonic drug treatment and image acquisition procedure

Drug treatment of *C. elegans* embryos was performed on permeabilized eggs. For this, young adult hermaphrodites were injected with *perm-1* dsRNA and incubated at 20°C for 16 h after the injection. *perm-1(RNAi)* embryos were than extruded from the gravid hermaphrodite in a solution of egg buffer or 20 mM arsenite (MerckMillipore) diluted in egg buffer or 20 mM arsenite plus 250 μg/ml cycloheximide (Sigma Life Sciences, C7698-1G; Lee et al., 2020) diluted in egg buffer on a 22×40 mm coverslips. The coverslips with dissected worms and embryos were incubated for 1 h at room temperature in a humid chamber.

After the incubation time, the coverslip was mounted on a 3% agarose pad for imaging. Imaging was performed using the Leica DM6000 described above. Images were acquired using the 63×/1.4 numerical aperture (NA) objective and the LAS AF software (Leica Biosystems). The percentage of granule-containing embryos was counted.

### Hermaphrodite heat shock, drug treatment and image acquisition procedure

For heat shock, young adult worms were transferred into a drop of M9 buffer (86 mM NaCl, 42 mM Na_2_HPO_4_, 22 mM KH_2_PO_4_ and 1 mM MgSO_4_) on a glass coverslip and transferred on a metal block placed into a humidified incubator for 10 min at 35°C. For recovery after heat shock, worms were collected from the M9 drop and transferred onto OP50 seeded NGM plates and incubated at 20°C for 5 or 10 min.

For drug treatment, young adult worms were transferred into a drop of M9 buffer only (control) or in 10 mg/ml of puromycin (InvivoGen) or with 20 mM arsenite (MerckMillipore). Worms were incubated in the puromycin-containing solution for 4 h and in the arsenite-containing solution for 5 h before imaging. Control worms were incubated in M9 buffer for the same amount of time as the puromycin or arsenite-treated worms.

Control and drug-treated worms were then transferred in a drop of NaN_3_ 30 mM (for worm paralysis) and mounted on a 3% agarose pad for imaging. Imaging was performed using the Leica DM6000 described above. Images were acquired using the 63×/1.4 numerical aperture (NA) objective and the LAS AF software (Leica Biosystems).

### Immunostaining of embryos and image acquisition procedure

For *C. elegans* embryos staining, 20–25 gravid hermaphrodites were dissected in a drop of M9 buffer on 22×40 mm coverslips. Control samples were left at room temperature (20–22°C) for 10 min. For heat shock exposure, coverslips with dissected worms and embryos, were transferred on a metal block placed in a humidified incubator at 34°C for 10 min. For recovery, after the heat shock, the coverslip was transferred on a metal block at room temperature (20°C).

After the incubation time, the coverslip was mounted crosswise on the epoxy slide square (Thermo Fisher Scientific), previously coated with 0.1% poly-L-lysine, for embryonic squashing. The slides were then transferred on a metal block on dry ice for at least 10 min. Afterward, the coverslip was removed (freeze-cracking method) before fixation. Immunostaining was performed as described in Spilker et al. (2009). Briefly, embryos were fixed for 20 min in methanol and placed for 20 min in a solution of PBS and 0.2% Tween 20 (PBST) and BSA 1% to block the nonspecific antibody binding. The slides were incubated with primary antibodies diluted in PBST with 1% BSA overnight at 4°C. The list of primary antibodies used in this study is in Table S5. After two washes of 10 min each in PBST, slides were incubated for 45 min at 37°C with a solution containing secondary antibodies (4 µg/ml Alexa Fluor 488- and/or 568-coupled anti-rabbit or anti-mouse antibodies from Molecular Probes) and 1 µg/ml DAPI to visualize DNA in PBST. Slides were then washed two times for 10 min in PBST before mounting using Mowiol [30% (w/v) glycerol, 3.87 mM Mowiol (Calbiochem, 475904), 0.2 M Tris-HCl pH 8.5, and 0.1% DABCO].

In the case of GFP- or RFP-tagged strains, the slides were briefly (10 min at room temperature) incubated with 1 µg/ml DAPI in PBST to visualize DNA just after methanol fixation and blocking. Slides were then washed two times for 10 min in PBST and mounted with Mowiol.

Images were acquired using a Nikon A1r spectral (inverted Ti Eclipse) confocal microscope equipped with a 60×1.4 NA CFI Plan Apochromat Lambda oil objective and four photomultiplier tubes (PMTs) including two highly sensitive detectors (GaAsp) for the green and red channels. Five to seven *z*-stacks, separated by 0.5 µm, were acquired. NIS Elements AR software (v.4.20.01; Nikon) was used to set acquisition parameters.

### Fluorescence *in situ* hybridization

The FISH protocol is adapted from Lee et al. (2020). FISH probes recognizing poly(A) tails of mRNA molecules were designed using Biosearch Technologies' Stellaris Probe Designer. The probe used in this study is a 30dT oligonucleotide coupled at the 3′ with the Quasar Q670 fluorophore.

For sample preparation, as described above, embryos were extruded from gravid hermaphrodites in a drop of M9 on 22×40 mm coverslips. Control samples were left at room temperature (20–22°C) for 10 min. For heat-shock exposure, coverslips with dissected worms and embryos, were transferred onto a metal block placed in a humidified incubator at 34°C for 10 min. After the incubation time, the coverslip was mounted crosswise on the epoxy slide square, previously coated with 0.1% poly-L-lysine, for embryonic squashing. The slides were then transferred on a metal block on dry ice for at least 10 min and subjected to freeze-crack, followed by cold methanol fixation at −20°C. For rehydration, samples were washed once with 0.5× PBS with 0.1% Tween 20 and methanol (1:1) and once with 0.5× PBS with 0.1% Tween 20, followed by five other washes in 1× PBS plus 0.2% Tween 20. Samples were subsequently fixed in 4% PFA (Sigma Life Sciences) diluted in PBS for 1 h at room temperature in a humid chamber. After PFA fixation, samples were again washed four times in 1× PBS with 0.2% Tween 20, twice in 2× SSC (0.3 M NaCl, 0.03 M sodium citrate), and once in wash buffer (10% formamide, 2× SSC). The blocking step was performed incubating the samples in Hybridization Buffer [10% formamide, 2× SSC, 200 µg/ml ultrapure BSA, 2 mM ribonucleoside vanadyl complex (NEBS, #S1402S), 0.2 mg/ml yeast total RNA (Roche, REF 10109223001), 10% dextran sulfate] for 30 min at 37°C in a humid chamber. For hybridization, samples were incubated overnight at 37°C with 50 nM probe solutions diluted in hybridization buffer. Samples were subsequently washed twice in wash buffer (each wash followed by an incubation of 30 min at 37°C), twice in 2× SSC, once in 1× PBS with 0.2% Tween 20, and twice in 1× PBS. Finally, samples were mounted using Mowiol mounting medium with 1× DAPI.

Images were acquired using a Nikon A1r spectral (inverted Ti Eclipse) confocal microscope, as described in Immunostaining of embryos and image acquisition procedure above.

### Quantification of cytoplasmic protein levels

The mean intensity of a defined region of interest (ROI) (w=2.69, h=2.46 pixels, area=6.604 pixels^2^), always placed in the anterior blastomere (AB) of a two-cell stage *C. elegans* embryo or in the last oocyte of a *C. elegans* germline, was measured using Fiji ImageJ. The mean intensity of an equal ROI, placed outside of the embryo, was used for background subtraction. For each experiment, the obtained mean intensity values were normalized on the highest value for 0 to 100 (%) scale conversion.

### Quantification of cytoplasmic granules

For the quantification of PQN-59, GTBP-1 and TIAR-1 embryonic cytoplasmic granules, QuPath version 0.2.3 was used (Bankhead et al., 2017). The algorithm for granule detection was based on a pixel classifier and was trained on representative pictures with dedicated annotations. For each manually delineated embryo, the total number of detected granules was obtained. The average intensity of all the detected granules in each embryo was background subtracted using the average embryonic intensity of the same embryo.

Germline granules of epifluorescence pictures in Figs 3, 4 and Fig. S4, were quantified using as a readout the standard deviation of the gray value of a defined region of interest (ROI) (w=214, h=64 pixels, area=9088 pixels^2^). Values were quantified in both the distal and proximal germline. A diffuse signal gives a smaller standard deviation while a punctate signal, as seen for stress granules, results in high standard deviation.

### Yeast two-hybrid assay

The interaction between PQN-59 and GTBP-1 was assessed in the PJ69-4a yeast strain (James et al., 1996) using single copy GAL4 activation and GAL4 DNA-binding domain-based vectors. Full-length cDNAs were cloned into these vectors using Gibson reactions and transformed into the host yeast strain using previously described protocols (James et al., 1996). Transformants were selected on SC −Leu −Trp plates and subsequently tested for growth (3 days) on SC −Leu −Trp −His plates containing 3 mM 3-amino-1,2,4-triazole (3AT).

### Protein domain identification

Protein domains were identified using the meta site Motif Scan tool, a free database for protein motif prediction developed by the Swiss Institute of Bioinformatics (SIB), including Prosite, Pfam, and HAMAP profiles (https://myhits.isb-sib.ch/cgi-bin/motif_scan). Comparable results were also obtained interrogating other online tools, such as PROSITE at ExPASy (https://prosite.expasy.org/), MOTIF (GenomeNet, Institute for Chemical Research, Kyoto University, Japan) (https://www.genome.jp/tools/motif/), and InterPro (http://www.ebi.ac.uk/interpro/).

Prion domains were identified using PLAAC (http://plaac.wi.mit.edu).

### Antibody production

To produce antibodies against PQN-59, a C-terminal fragment (amino acids 304–712) was cloned using the Gateway technology (Invitrogen) into the pDEST15. Recombinant GST-tagged PQN-59 was expressed in BL21 and purified using standard protocols. Antibody production in rabbit was performed by Covalab, France. The obtained anti-PQN-59 serum was purified on membrane strip carrying bacterially expressed GST–PQN-59 antigen. Approximately 5 µg of fusion protein was loaded in each lane of a 10% acrylamide gel. The protein was transferred on a nitrocellulose membrane (GE Healthcare). A stripe of the membrane, containing the protein, was cut and incubated for 1 h in PBS plus 3% milk for blocking. The band was then incubated overnight at 4°C in 1 ml of serum diluted in 1 ml of 3% milk in PBS with 0.01% Tween 20 plus 4 mg of GST (to compete for a GST antibody binding). After three washes of 5 to 10 min, the antibody was eluted using a solution of glycine 100 mM, pH 2. The pH of the elution solution was equilibrated to 7.5 using 1 M Tris.

### Western blotting

For western blotting, 50 adult worms were manually picked from NGM plates, resuspended in Laemmli sample buffer and incubated at 92°C for 2 min. Lysates were separated by SDS-PAGE using a 10% acrylamide gel. Proteins were then transferred onto a nitrocellulose membrane (Sigma). The membrane was blocked with 3% milk dissolved in PBS and 0.01% Tween 20 (PBST). After washing with PBST, the membrane was incubated overnight at 4°C with primary antibodies diluted in a 1% BSA PBST solution (see Table S5). The following day the membrane was washed with PBST twice for 10 min and incubated with secondary antibodies diluted in the same solution [1:10,000 HRP-conjugated anti-mouse or anti-rabbit antibodies (Biorad)] at room temperature for 45 min. After three washes of 10 min each, proteins were visualized with ECL (Millipore) using a Pxi (Syngene) machine.

### Embryonic lethality and brood size counting

To count embryonic lethality and the number of laid eggs, L4 or young adult worms were singled onto individual OP50-seeded NGM plates and incubated at 20°C for 24 h. After 24 h, the adult worm was removed, and plates were again incubated at 20°C for 24 h. To assess the total number of laid eggs, the number of non-hatched embryos and hatched larvae were counted under a dissecting microscope. The ratio between the non-hatched embryos over the total of the progeny was used to calculate the percentage of embryonic lethality.

To count the total brood size, as shown in Fig. 7C, L4 animals were manually transferred to individual NGM plates seeded with OP50 bacteria. Adults were manually transferred onto new OP50-seeded NGM plates every 24 h until no more eggs were laid. The number of eggs laid and larvae present on each plate was counted 24 h after the removal of the adult worm. The number of eggs laid and larvae counted every 24 h was finally summed up to assess the total brood size.

### Embryonic lethality after heat shock

For embryonic lethality after heat shock, gravid hermaphrodites were dissected on a coverslip into a drop of egg buffer where the embryos were released. The coverslip was then transferred on a metal block placed in a humidified incubator at 34°C for 10 min. After the heat shock, the embryos were transferred by pipetting on OP50-seeded NGM plates, counted, and incubated at 20°C for 24 h for recovery. After recovery, the number of non-hatched embryos was counted. The ratio between non-hatched embryos over the number of embryos plated was used to calculate the percentage of embryonic lethality after heat shock exposure.

### Statistical analysis

Statistical analysis was performed using GraphPad Prism 8. Details on the statistical test, the sample, and experiment number, as well as the meaning of error bars, are provided for each experiment in the corresponding figure legend, in the results and/or in the method details. Significance was defined as: ns, not significant; *P*>0.05; ^∗^*P*<0.05; ^∗∗^*P*<0.01; ^∗∗∗^*P*<0.001; ^∗∗∗∗^*P*<0.0001.

## Supplementary Material

Supplementary information

Reviewer comments
