# Supplementary material for: PQN-59 and GTBP-1 contribute to stress granule formation but are not essential for their assembly in C. elegans embryos
Source: J Cell Sci. 2021 Nov 15;134(22):jcs258834. doi: 10.1242/jcs.258834 (PMC8645233; doi:10.1242/jcs.258834)
Supplement: Supplementary information [file joces-134-258834-s1.pdf]

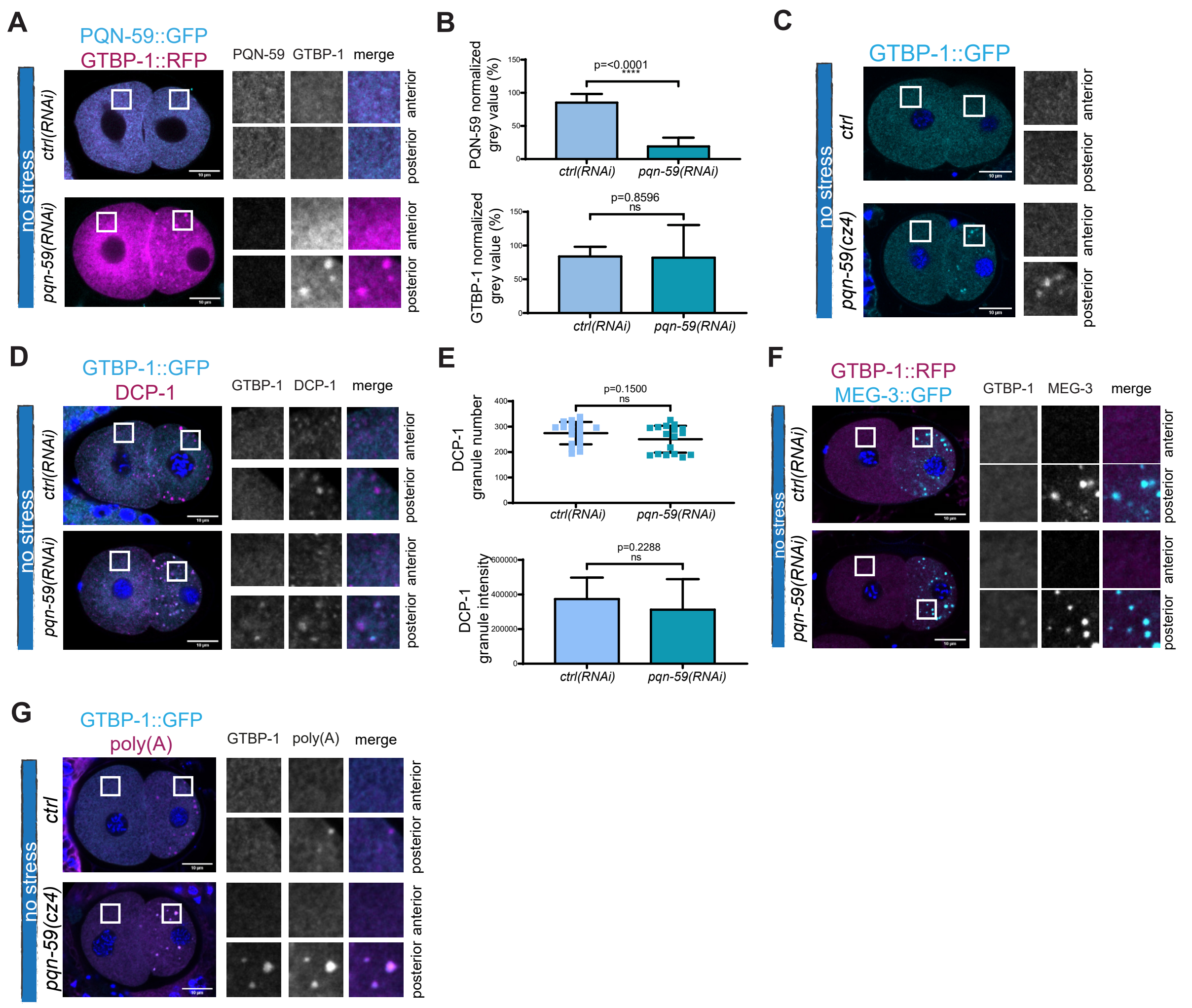

Fig. S1. PQN-59 RNAi depletion or deletion results in stress-independent GTBP-1 granule formation in the posterior P1 blastomere. Related to Fig.3

(A) Single confocal planes of *pqn-59::GFP;gtbp-1::RFP* fixed two-cell stage embryos treated with the indicated RNAi in non-heat-shocked conditions (no stress). (B) Quantification of the normalized cytoplasmic intensity of PQN-59 (top) and GTBP-1 (bottom) of *ctrl(RNAi)* and *pqn-59(RNAi)* in the anterior blastomere as in (A) (*ctrl(RNAi)* n=23; *pqn-59(RNAi)* n=38, N=4). (C) Single confocal planes of fixed two-cell stage *gtbp-1::GFP* and *pqn-59(cz4);gtbp-1::GFP* embryos, at 20°C (no stress). GTBP-1 GFP signal is in cyan and DNA was counterstained with DAPI (blue). (*ctrl* n=16, *pqn-59(cz4)* n=11, N=2). **(D)** Single confocal planes of *gtbp-1::GFP* fixed two-cell stage embryos (no stress) immunostained with DCP-1 antibodies (magenta). GTBP-1 GFP signal is in cyan and DNA was counterstained with DAPI (blue). Embryos were treated with *ctrl* or *pqn-59(RNAi)* as indicated (*ctrl(RNAi)* n=19; *pqn-59(RNAi)* n=17, N=3). (E) Quantifications of the number (top) and intensity (bottom) of DCP-1 granules in control and *pqn-59(RNAi)* embryos (from D). (F) Single confocal planes of *gtbp-1::RFP; meg-3::GFP* control and *pqn-59(RNAi)* two cell embryos not exposed stress. (G)

Journal of Cell Science • Supplementary information

Single confocal planes of fixed two-cell stage *gtbp-1::GFP* and *pqn-59(cz4);gtbp-1::GFP* embryos (no stress) hybridized with a poly(A) FISH probe. GTBP-1 GFP signal is in cyan, the poly(A) signal in magenta and DNA was counterstained with DAPI (blue). (*ctrl* n=11, *pqn-59(cz4)* n=16, N=2) Error bars in B and E indicate S.D. The P-value was determined using two-tailed unpaired Student's *t*-test. n indicates the number of samples and N the number of independent experiments. ROIs are shown enlarged on the right of each set of embryos. Scale bars represent 10 μm.

Journal of Cell Science • Supplementary information

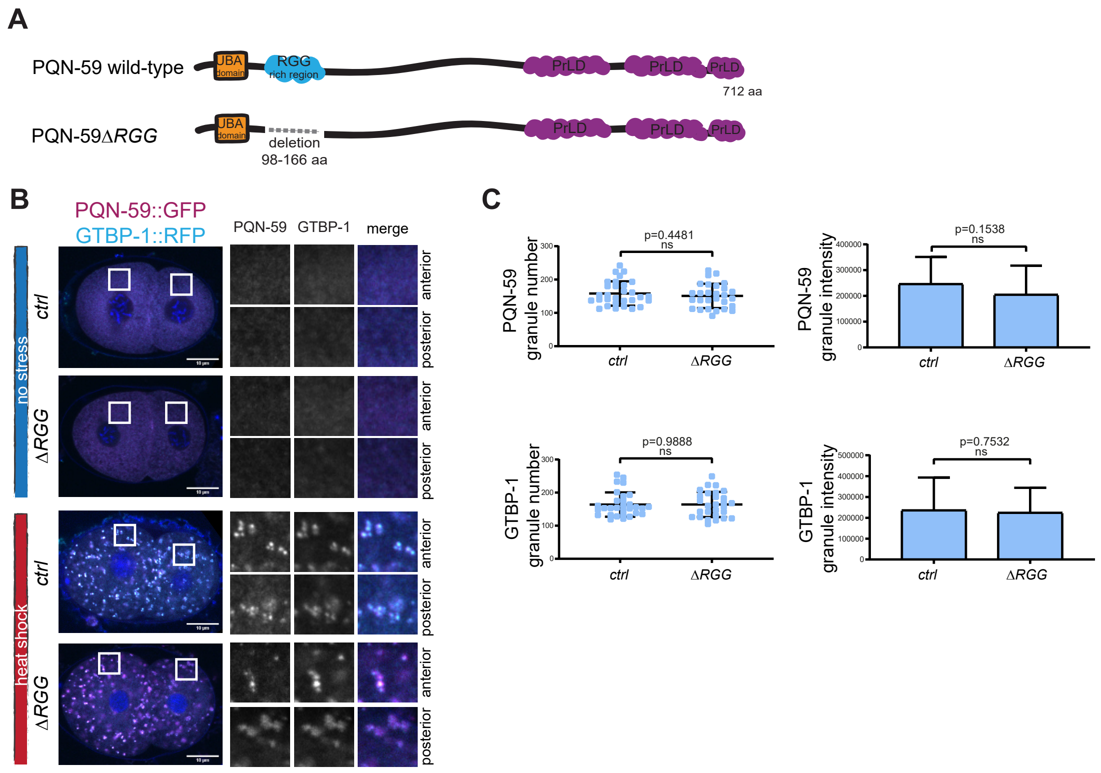

Fig. S2. The RGG domain of PQN-59 is not required to assemble stress granules.

(A) Illustration of the PQN-59 protein with the RGG deletion introduced by CRISPR/Cas9 in the strain *pqn-59::GFP;gtbp-1::RFP*. (B) Single confocal planes of *pqn-59::GFP;gtbp-1::RFP* and *pqn-59::ΔRGG::GFP;gtbp-1::RFP* fixed two-cell stage embryos. PQN-59 GFP signal is in cyan, GTBP-1 RFP signal is in magenta and DNA was counterstained in blue. Scale bars represent 10 µm. (C) Quantification of the average PQN-59 (top left) and GTBP-1 (bottom left) granule number per embryo and of the average normalized PQN-59 (top right) and GTBP-1 (bottom right) granule intensity per embryo (*pqn-59::GFP;gtbp-1::RFP* n=30; *pqn-59::ΔRGG::GFP;gtbp-1::RFP*, n=28, N=3). Error bars indicate S.D. The P-value was determined using two-tailed unpaired Student's *t*-test. n indicates the number of samples and N the number of independent experiments.

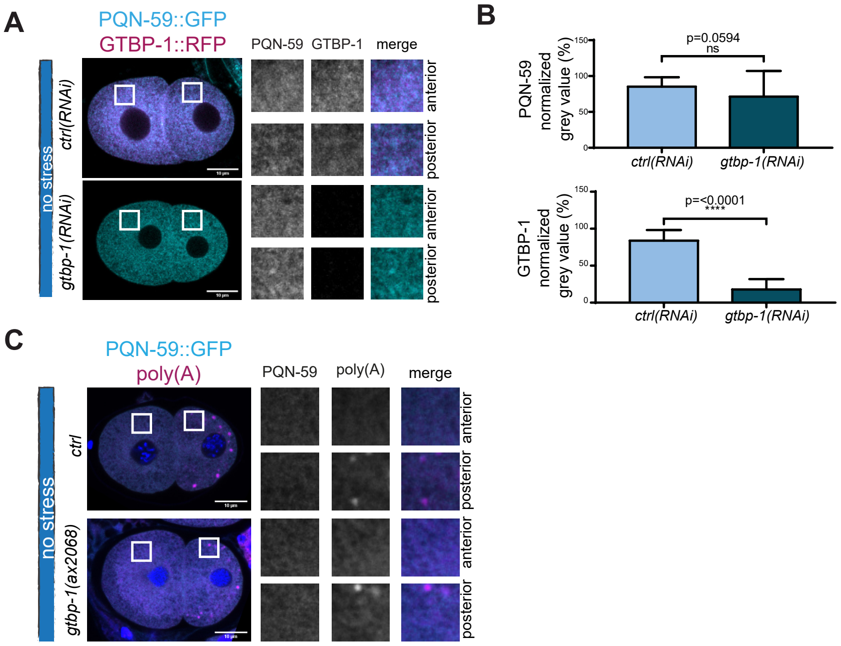

Fig. S3. PQN-59 localization and levels in control and *gtbp-1(RNAi)* or *gtbp-1(ax2068)* embryos not exposed to stress. Related to Fig.4

(A) Single confocal planes of *pqn-59::GFP;gtbp-1::RFP* fixed two-cell embryos treated with the indicated RNAi depletion in non-heat-shocked conditions (no stress). (B) Quantification of the normalized cytoplasmic intensity of PQN-59 (top) and GTBP-1 (bottom) of *ctrl(RNAi)* and *pqn-59(RNAi)* embryos at 20°C as in A (*ctrl(RNAi)* n=18; *gtbp-1(RNAi)* n=34, N=4). Error bars indicate S.D. The P-value was determined using Student's t-test. (C) Single confocal planes of *pqn-59::GFP* and *pqn-59::GFP;gtbp-1(ax2068)* fixed two-cell embryos hybridized with a poly(A) FISH probe. *ctrl*, n= 21, *gtbp-1(ax2068)*, n=16, N=2. n indicates the number of samples and N the number of independent experiments. Enlarged ROIs are shown on the right. Scale bars represent 10 µm.

Journal of Cell Science • Supplementary information

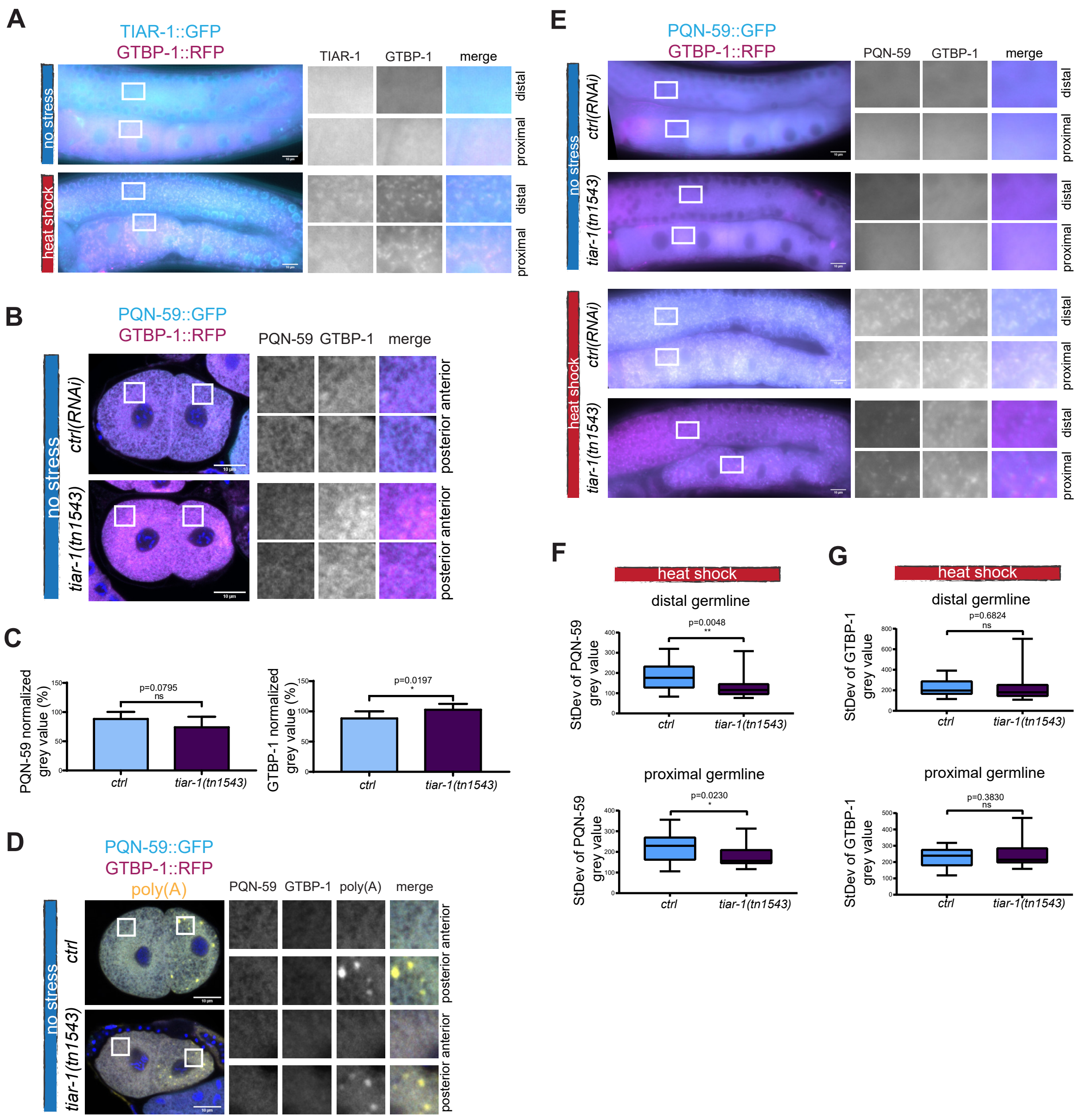

Fig. S4. TIAR-1 localization in germlines exposed to heat shock and PQN-59 and GTBP-1 localization in *tiar-1(tn1543)* mutant embryos and germlines. Related to Fig. 5 and 6

(A) Epifluorescence images of *tiar-1::GFP;gtbp-1::RFP* germlines. TIAR-1 (in cyan) and GTBP-1 (in magenta) assemble into granules that colocalize in germlines exposed to heat shock (no stress, n= 26 and stress, n= 34). (B) Single confocal planes of *pqn-59::GFP;gtbp-1::RFP* fixed control and *tiar-1(tn1543)* two-cell embryos. PQN-59 is in cyan and GTBP-1 in magenta (C) Quantification of the PQN-59 (left) and GTBP-1 (right) levels as in B. For PQN-59 levels, *ctrl*, n=7, *tiar-1(tn1543)*, n=7. For GTBP-1 levels, *ctrl*, n=7, *tiar-1(tn1543)*, n=7, N=1. (D) Single confocal planes of *pqn-59::GFP;gtbp-1::RFP* fixed control (n=9) and *pqn-59::GFP; tiar-1(tn1543);gtbp-1::RFP* (n=7) two-cell embryos hybridized with a poly(A) FISH probe. N=2. PQN-59 is in cyan, GTBP-1 in magenta and the poly(A)

signal in yellow. (E) Germline pictures of *pqn-59::GFP;gtbp-1::RFP* control (n=10, no stress, n= 24, HS) and *tiar-1(tn1543)* (n=10, no stress, n= 23, HS) taken with epifluorescence microscope exposed (bottom) or not (top) to heat shock. PQN-59 is in cyan and GTBP-1 in magenta. (F) and (G) Quantification of the standard deviation of the PQN-59 and GTBP-1 grey values in *control* and *tiar-1(tn1543)* distal (top) and proximal (bottom) germlines, sample number as in E. Error bars indicate S.D. The P-value was determined using two-tailed unpaired Student's *t*-test. n indicates the number of samples and N the number of independent experiments. Enlarged ROIs are shown on the right. Scale bars represent 10 µm.

Table S1. Strain List. The strain used in this work are listed in this table in order of appearance in the text. The genotype, source and description of the mutation are also reported.

Strain name	Genotype	Source/Reference	Description
**HML713**	*csIs25(pqn-59::GFP) I;gtbp-1::RFP(ax5000) IV*	(Carlston et al., 2021 preprint)	Strain generated by C. Hammell using CRISPR/Cas9. GFP has been inserted at the C-terminal of PQN-59 on the genotype K08F4.2 *(ax5000[gtbp-1::tagRFP]) IV.*
**JH3199**	*gtbp-1(ax2055[gtbp-1::GFP]) IV*	CaenorhabditisGenetics Center (CGC)	
**ZU291**	*pqn-59::ollas::STOP(cz4);gtbp-1(ax2055[gtbp-1::GFP]) IV*	This study	Strain generated by CRISPR/Cas9, inserting a frameshift mutation generating a premature STOP. Background strain is JH3199 (Paix et al., 2014).
**ZU304**	*gtbp-1::tagRFP(ax5000) IV;meg-3(ax3054[meg-3::meGFP]) X*	This study	Strain obtained crossing the JH3503 (*meg-3::GFP*) (Smith et al., with the *gtbp-1::tagRFP.*
**ZU288**	*pqn-59::ΔRGG::GFP;gtbp-1::RFP(ax5000)* – clone 1	This study	Strain generated by CRISPR/Cas9, excising the RGG region of PQN-59. Background strain is HML713.
**HML275**	*csIs25(pqn-59::GFP) I*	(Carlston et al., 2021 preprint)	Strain generated by C. Hammell using CRISPR/Cas9. GFP has been inserted at the C-terminal of PQN-59 in a N2 background.
**HML703**	*csIs25(pqn-59::GFP) I; gtbp-1(ax2068)*	(Carlston et al., 2021 preprint)	Strain generated by C. Hammell crossing the JH3212 (bearing a 1.6 kb deletion in *gtbp-1,* (Paix et al., 2014)) with the HML275.
**JH3176**	*gtbp-1(ax2029) IV*	CaenorhabditisGenetics Center (CGC)<br>(Paix et al., 2014)	Mutant bearing a STOP codon insertion and frameshift mutation near the ATG of *gtbp-1.*

Journal of Cell Science • Supplementary information

**DG3922**	*tiar-1(tn1545[tiar-1::s::tev::GFP]) II*	CaenorhabditisGenetics Center (CGC)<br><br>(Huelgas-Morales et al., 2016)	
**ZU294**	*tiar-1(tn1545[tiar-1::s::tev::GFP]) II; gtbp-1(ax2029) IV*	This study	Strain obtained crossing the DG3922 with the JH3176.
**ZU306**	*csIs25(pqn-59::GFP) I; tiar-1(tn1543)(loxP:Cbr-unc-119(+)::loxP)) II; gtbp-1::RFP(ax5000) IV*	This study	Strain obtained crossing the DG3929 (TIAR-1 mutant generated by CRISPR/Cas9 (Huelgas-Morales et al., 2016)) with the HML713.
**ZU287**	*tiar-1(tn1545[tiar-1::s::tev::GFP]) II; gtbp-1::tagRFP(ax5000)*	This study	Strain obtained crossing the DG3922 with the genotype K08F4.2 *(ax5000[gtbp-1::tagRFP]) IV.*
**N2**	*wild type*	CaenorhabditisGenetics Center (CGC)	
**ZU278**	*pqn-59::ollas::STOP(cz2)*	This study	Strain generated by CRISPR/Cas9, inserting a frameshift mutation generating a premature STOP. Background strain is N2.

Journal of Cell Science • Supplementary information

Table S2. CRISPR reagents.

Strain name	sgRNA sequence (5'->3')			Repair template sequence	Description
**ZU291**	1	FW<br>TCT TGG AAC AGA AAG GAG CCA AAA	RV<br>AAA CTT TTG GCT CCT TTC TGT TCC	TGG AGA TAA ACT TGA CTC GTG GAC TGA ACA GAA AGG AGC CAA AA**A AG**A AAA GAA GAA G*tc cgg att cgc caa cGA GCT Cgg acc acg tct cat ggg aaa g*C**C TG**A A~~*T*A G~~GG CAG T~~TA *A*~~AA CAA CAG AGG ATT TGT AGC AAG AGG CAG AG	Insertion of the *ollas tag* for screening +<br>Nucleotide ***substitution*** generating a ~~prematureSTOP~~
	2	FW<br>TCT TGA AAA AGG AAA AGA AGA AGC	RV<br>AAA CGC TTC TTC TTT TCC TTT TTC		
**ZU288**	1	FW<br>TCT TGG AGG GCA GTT ATA ACA ACA G	RV<br>AAA CCT GTT GTT ATA ACT GCC CTC C	AAGGAGCCAAAAAGGAAAAG AAGAAGCCGGAAGAGGGCAG TTATAACAAC ~~*deletion*~~ TATTCTCGAGCTGTTGCTCCAT CATCAGCACTTGAGCCAGATG CGTTCAC	Deletion of the RGG domain (from aa 98 to aa 166)<br>(PAM sites are inside the deleted region, therefore not shown in the repair template sequence)<br>(clone 1)
	2	FW<br>TCT TG G CTC GAG AAT ATC CAC CTC	RV<br>AAA CGA GGT GGA TAT TCT CGA GCT C		
**ZU278**		FW<br>TCT TGG AAC AGA AAG GAG CCA AAA	RV<br>AAA CTT TTG GCT CCT TTC TGT TCC	TGGAGATAAACTTGACTC GTGG***G***AC~~TGA~~ACAGAAAG GAGCCAAAA**AAG**AAAAGA AGAAG*tccggattcgccaacG AGCTCggaccacgtctcatgggaa ag*C**CTG**AAGAGGGCAGTT ATAACAACAGAGGATTTG TAGCAAGAGGCAGAG	G nucleotide ***insertion*** + frameshift +<br>*ollas tag* for screening + ~~prematureSTOP~~
	1	FW<br>TCT TGA AAA AGG AAA AGA AGA AGC	RV<br>AAA CGC TTC TTC TTT TCC TTT TTC		

Journal of Cell Science • Supplementary information

LABEL

Underlined sequence is targeted by the sgRNA

In capital bold the silently mutated **PAM sites** (the first one originally AGG mutated in AAG and the second one originally CGG mutated in CTG)

In italic is the *OLLAS* sequence containing a SacI restriction site (italic upper cases)

In bold capital italic the ***nucleotide substitution*** or ***insertion***

Strikethrough in the sequence are the premature ~~STOP codon~~ and the ~~deletion~~

Journal of Cell Science • Supplementary information

Table S3. Oligonucleotides for PCR genotyping and sequencing.

Strain	Gene		Oligonucleotide sequence		Description
			FW primer (5'->3')	RV primer (5'->3')	
**ZU291 and ZU278**	*pqn-59*	1	ttctagtctagcttgcggtg	GCACCGATCTCATTTGCTG	PCR product is ~1600bp. After SacI digestion of this PCR product, fragments of ~1100bp and ~500bp are generated in the mutant.
	pqn-59	2	accacgtctcatgggaaag	GCACCGATCTCATTTGCTG	FW primer is annealing to the ollas sequence. PCR product is ~1100bp.
**ZU288**	*pqn-59*	1	CCAAAAAGGAAAAGAAGAAGC	GGATGCTGTTGTGGATGTCC	The primers are external to the deletion. In the mutant, the PCR product is ~1300bp.
		2	CCAAAAAGGAAAAGAAGAAGC	GGATGCTGTTGTGGATGTCC	The FW primers is internal to the deletion. In the mutant, there is no amplification.
**JH3176**	*gtbp-1*		ctttcgaatttcgcgcgttc	GAACCTCCTCGATTTCTCC	PCR product is ~1600bp. After NheI digestion of this PCR product, fragments of ~900bp and ~700bp are generated in the mutant.

Journal of Cell Science • Supplementary information

Table S4. dsRNA sequences.

Target gene	Clone name from Ahringer library	Oligonucleotides sequence (5'->3')	
ctrl	C06A6.2	Standard T7 primers	
pqn-59		FW<br>CCAAATCAAGCAT<br>GGACCA	RV<br>TTA GTT ACT CCA GTT GTA CG
gtbp-1	K08F4.2	Standard T7 primers	
tiar-1		FW<br>CGTAATACGACTCACT<br>ATAGcagGAGATGAAAGTCA<br>ACTG	RV<br>CGTAATACGACTCA<br>CTATAGtacCAGTAAGTGAAGC<br>AATG
perm-1	*T01H3.4*	Standard T7 primers	

Table S5. Primary antibodies list.

Antibody	Source/Reference	Working concentration/dilution	
		IF	WB
**rabbit anti-PQN-59**	This study	2ng/μl	2ng/μl
**rabbit anti-DCP-1**	Gift from Jayne Squirrell<br>(Squirrell et al., 2006)	1:5000	
**Mouse anti-a TUBULIN**	Sigma-Aldrich		1:2500

References

Carlston, C., Weinmann, R., Stec, N., Abbatemarco, S., Schwager, F., Wang, J., Ouyang, H., Gotta, M., Hammell, C.M., 2021. PQN-59 antagonizes microRNA-mediated repression and functions in stress granule formation during C. elegans development. BiorXiv 2021.05.14.444139; doi: https://doi.org/10.1101/2021.05.14.444139

Huelgas-Morales, G., Silva-Garcia, C. G., Salinas, L. S., Greenstein, D. & Navarro, R. E. The Stress Granule RNA-Binding Protein TIAR-1 Protects Female Germ Cells from Heat Shock in Caenorhabditis elegans. *G3 (Bethesda)* **6**, 1031-1047, doi:10.1534/g3.115.026815 (2016).

Paix, A. *et al.* Scalable and versatile genome editing using linear DNAs with microhomology to Cas9 Sites in Caenorhabditis elegans. *Genetics* **198**, 1347-1356, doi:10.1534/genetics.114.170423 (2014).

Smith, J. *et al.* Spatial patterning of P granules by RNA-induced phase separation of the intrinsically-disordered protein MEG-3. *Elife* **5**, doi:10.7554/eLife.21337 (2016).

Squirrell, J. M. *et al.* CAR-1, a protein that localizes with the mRNA decapping component DCAP-1, is required for cytokinesis and ER organization in Caenorhabditis elegans embryos. *Mol Biol Cell* **17**, 336-344, doi:10.1091/mbc.e05-09-0874 (2006).
