# Supplementary material for: PQN-59 and GTBP-1 contribute to stress granule formation but are not essential for their assembly in C. elegans embryos
Source: J Cell Sci. 2021 Nov 15;134(22):jcs258834. doi: 10.1242/jcs.258834 (PMC8645233; doi:10.1242/jcs.258834)
Supplement: Reviewer comments [file joces-134-258834_review_history.pdf]

PQN-59 and GTBP-1 contribute to stress granule formation but are not essential for their assembly in *C. elegans embryos*

Simona Abbatemarco, Alexandra Bondaz, Francoise Schwager, Jing Wang, Christopher M. Hammell and Monica Gotta
DOI: 10.1242/jcs.258834

Editor: Maria Carmo-Fonseca

Review timeline

Original submission:	23 April 2021
Editorial decision:	24 May 2021
First revision received:	3 September 2021
Editorial decision:	30 September 2021
Second revision received:	6 October 2021
Accepted:	9 October 2021

Original submission

First decision letter

MS ID#: JOCES/2021/258834

MS TITLE: The UBAP2L ortholog PQN-59 contributes to stress granule assembly and development in *C. elegans*

AUTHORS: Simona Abbatemarco, Alexandra Bondaz, Francoise Schwager, Jing Wang, Christopher M. Hammell, and Monica Gotta
ARTICLE TYPE: Research Article

We have now reached a decision on the above manuscript.

To see the reviewers' reports and a copy of this decision letter, please go to: https://submit-jcs.biologists.org and click on the 'Manuscripts with Decisions' queue in the Author Area. (Corresponding author only has access to reviews.)

As you will see from their reports, the reviewers' recommendations regarding publication are somewhat mixed. While referee #1 considers that your study represents too little progress for warranting publication, the other reviewers thought that the work was potentially quite interesting and significant but all also raised a number of concerns that must be dealt with. If you think that you can deal satisfactorily with the criticisms on revision, namely if you can perform the additional experiments suggested by referees #2 and #3, I would be pleased to see a revised manuscript. I would then return it to the reviewers.

We are aware that you may be experiencing disruption to the normal running of your lab that makes experimental revisions challenging. If it would be helpful, we encourage you to contact us to discuss your revision in greater detail. Please send us a point-by-point response indicating where you are able to address concerns raised (either experimentally or by changes to the text) and where you will not be able to do so within the normal timeframe of a revision. We will then provide further guidance. Please also note that we are happy to extend revision timeframes as necessary.

© 2021. Published by The Company of Biologists under the terms of the Creative Commons Attribution License (https://creativecommons.org/licenses/by/4.0/).

Please ensure that you clearly highlight all changes made in the revised manuscript. Please avoid using 'Tracked changes' in Word files as these are lost in PDF conversion.

I should be grateful if you would also provide a point-by-point response detailing how you have dealt with the points raised by the reviewers in the 'Response to Reviewers' box. Please attend to all of the reviewers' comments. If you do not agree with any of their criticisms or suggestions please explain clearly why this is so.

Reviewer 1

Advance summary and potential significance to field

This manuscript from the Gotta lab investigates two C. elegans orthologs of proteins with roles in mammalian stress granule assembly. The authors demonstrate that heat stress induces PQN-59/UBAP2L and GTBP-1/G3BP1/2 to condense into granules in early embryos and into granule-like fibrils in the germ line. They also demonstrate that oxidative stress induces PQN-59 and GTBP-1 granule assembly in the germ line, and puromycin treatment (to inhibit translation) also promotes granules to a lesser extent. Unfortunately, the bulk of the remaining experiments have "negative results". The authors show that neither protein is strictly required for the other to condense into heat stress-induced granules, nor is either protein essential for TIAR-1 to form stress-induced granules in embryos (though there are modest effects shown). The RGG domain that nucleates mammalian UBAP2L stress granules is not required for PQN-59 granules in embryos, and neither PQN-59 nor GTBP-1 appear to have a role in mediating embryo survival during heat stress. While a negative result can oftentimes be informative, in this case the series of results fails to provide insight into any critical function of either protein. At the end of the manuscript, the authors also briefly explore roles of the two proteins in reproduction, independent of stress conditions. They show that depletion of PQN-59 but not GTBP-1, results in reduced brood size and embryonic lethality; however, no follow-up experiments are included to probe the basis for the reduced brood size or embryonic lethality.

The experiments that are presented appear to have been done quite rigorously. For example, antibody staining experiments validate several of the GFP-reporter experiments, and mutations are used to complement RNAi knock-down experiments.
Unfortunately, two main problems prevent this work from being of high significance to the field: one, the large number of negative results preclude new mechanistic insights from being revealed; and two, most of the experiments were designed without taking full advantage of the literature, making it difficult to integrate any understanding of PQN-59 and GTBP-1 with what is known about the many RNA-binding proteins that condense into granules in the C. elegans germ line in response to stress. In addition, some of the more interesting aspects of the results are not discussed or included in conclusions. However, even with a revised discussion, I do not think the experiments in this study make a significant contribution to the field of cell biology.

Comments for the author

Problems/limitations of the study

1. In Fig. 1, the authors use reporters strains for PQN-59 and GTBP-1 to show that heat stress induces granules in early embryos and in the germ line. The "granules" in the oocytes are more fibrillar than those in the embryo, and appear quite distinct from the large granules of RNA-binding proteins induced by meiotic arrest in oocytes (Jud et al., 2008; Noble et al., 2008; Huelgas-Morales et al 2016; several other refs). They also seem to differ from the granules induced by heat stress, osmotic stress, and anoxia (Jud et al., 2008; Huelgas-Morales et al 2016). The authors do not make any comparison between the fibrillar PQN-59 and GTBP-1 heat stress-induced granules, and those described in the literature. One simple question is whether PQN-59 or GTBP-1 co-localize with any of the RNA-binding proteins.

2. Some of the description in the text does not seem to match the figures.
For example, in Fig. S2B, PQN-59 and GTBP-1 do not seem co-localized in the proximal oocytes at all, and there is no mention of the large GTBP-1-only granules?

© 2021. Published by The Company of Biologists under the terms of the Creative Commons Attribution License (https://creativecommons.org/licenses/by/4.0/).

3. The overall flow of the results is a bit confusing: the questions being asked are about stress responses, yet many figures of data are shown without stress. No explanation or rationale is provided for why those questions or comparisons might be interesting. In fact, it appears that PQN-59 inhibits GTBP-1 granules in no-stress conditions, but promotes GTBP-1 granules during heat stress. There is no discussion of these opposite effects.

4. In several places, comments are made about changes in the size of granules, but no quantitation is presented regarding granule size, and the images do not present obvious changes.

5. In figure 2, there are very interesting differences in the anterior vs. posterior blastomere. This is acknowledged but not probed or discussed. Could the posterior granules be P granules? Do they co-localize with P granule markers?

6. I am not sure I am convinced the granules being studied are stress granules. The authors do not show effects of any stress besides heat stress on embryos; the homology between PQN-59 and UBAP2L is not very strong; PQN-59 (including its RGG domain) is not required to nucleate the granules – different from UBAP2L; in pqn-59(RNAi) embryos GTBP-1 co-localizes with a P-body marker; the heat stress-induced granules previously described in the literature contain several P-body proteins.

7. In Fig. 5, it is not clear why the brood size of wild type is so low?

8. PQN-59 appears to have an important role in reproduction (Fig. 5).
However, the rationale to explore this question was not really provided, and this section of the paper seemed very separate from the focus on stress. It seems straight-forward to probe why PQN-59 affects brood size – are there problems with oocytes, sperm, fertilization, mitosis of the stem cells, increased apoptosis?
Similarly, what type of embryonic lethality was observed? Is there an early or synchronous cell cycle arrest? Addressing more of these questions might allow the authors to gain insight into PQN-59 function.

9. The discussion is short and largely repeats the results because there really isn’t much to say about the bulk of the data. I do not agree with the interpretation of the effect of depleting PQN-59 and GTBP-1 on TIAR-1 granules.
There was no change in the number of granules, yet the authors conclude the two proteins have a role in TIAR-1 granule assembly.

10. The organization of the figures is difficult to understand, including the decision to have so many supplemental figures. Some seems to be important to the main story, while others could potentially be omitted. The titles of several suppl. figures do not work because panel A is not at all related to the question in panel B, e.g. SFig.4, SFig.6, SFig.7.

Minor comments
1. Some of the writing is imprecise, eg. PQN-59 “fell into” granules.

<u>Reviewer 2</u>

Advance summary and potential significance to field

Stress granules are organelles that are formed by liquid-liquid phase separation. They have been extensively studied in mammals however in C. elegans there is still so much to learn about how they are formed, what proteins and mRNAs associated with them and their functions. Some of the best know proteins that nucleated stress granules formation in mammals are G3BP1 and G3BP2 and UBAP2L. In this paper Abbatemarco et al. studied GTBP-1 and PQN-59, the homologs of G3BP1 and G3BP2 and UBAP2L, respectively. They showed that these proteins associated to stress granules in embryos and gonads during different stress conditions. Unexpectedly, neither GTBP-1 nor PQN-19 are essential for stress granules formations. Although their absence did have an effect on stress granules appearance. Experiments are well performed and the paper has important contributions to worm stress granules composition by adding two new components GTBP-1 and PQN-19. It is intriguing that these proteins are not essential for stress granules formation. It is possible that the

© 2021. Published by The Company of Biologists under the terms of the Creative Commons Attribution License (https://creativecommons.org/licenses/by/4.0/).

heat shock was too short and the authors should try to challenge the system with longer stress conditions. It will be important to test if stress granules are still formed if two stress granule components are silenced at the same time. At its present form this paper does not make a significant contribution to understand the function of stress granules.

Comments for the author

1. The paper should be reorganized in a way that it is easier for the readers to follow. There are many supplementary figures that should be main figures. It is hard to follow going back and forward from supplementary figures to main figures several times.

2. It is intriguing that pqn-59::gfp and gtbp-1::rfp granules are still present in tiar-1(RNAi) embryos. Ii would be interesting to see if the same results are observed in tiar-1 mutant animals.

3. TIAR-1 is essential for stress granules formation in the gonad. Authors should show if in tiar-1 mutant animals GTBP-1 and PQN-19 granules are still present.

Minor points:
-Fig 1D and E. Insets in no heat shock conditions do not have good resolution.
They just look like green and red squares.
-Fig 3A. Insets in GTBP-1::RFP do not have good resolution. They just black squares. There is a similar problem in several figures.
-There is a hole between blastome AB and P1 in the middle of the heat shock embryo in pqn-59(cz4);gtbp-1::GFP picture. Could authors present a better picture?
-Page 6, line 146. The depletion of PQN-59….
Do authors refer to silencing as depletion? It is confusing.
-Figure S3C. There are more DCP-1 granules in the pqn-59(RNAi) embryo than in the control. Is that correct?

Reviewer 3

Advance summary and potential significance to field

In this study Abbatemarco et al use C. elegans embryos and germ cells to investigate the mechanisms controlling dynamics of stress granules (SGs), membraneless RNA/protein assemblies that form in response to various stresses. The authors focus on two conserved proteins that are well-characterized SG nucleators in yeast and human cells: G3BP/GTBP-1 and UBAP2L/PQN-59. The authors find that GTBP-1 and PQN-59 are dispensable for SG formation. Granule morphology and disassembly , however, is altered following depletion/mutation of either protein.

This paper is reminiscent of a paper published last year in JCS that found that SGs in Drosophila also do not require canonical SG nucleators for their formation (Buddika et al., 2020). Unlike the Buddika study, the Abbatemarco only reports on three stress granule components and did not address whether recruitment of RNA to SGs is affected in the mutants. Nevertherless, this study is significant in providing a second example of an animal model where the “rules” of stress granule assembly from tissue culture do not appear to apply.
The authors also show that neither G3BP nor PQN-59 depleted embryos are hyper sensitized to heat stress but the significance of this finding is not clear since these embryos still assemble stress granules (albeit with altered morphology).

Comments for the author

While not essential for assembly, depletion of G3BP or PQN-59 does affect SG morphology
(I suggest the authors include a table to summarize these findings, as I found them difficult to keep track of).
The observations are mostly descriptive so it was difficult to draw any firm conclusions. Further examination of the SG defects in the absence of PQN-59 or G3BP or both may help draw firmer conclusions. I suggest the authors address at least one of the following questions 1) which protein is required to recruit RNA? is PQN-59 primarily responsible for recruiting G3BP to the granules but not

© 2021. Published by The Company of Biologists under the terms of the Creative Commons Attribution License (https://creativecommons.org/licenses/by/4.0/).

essential for other aspects of granule formation? what is the significance of the accelerated dissolution observed in the mutants?

1. The authors document that loss of PQN-59 causes altered recruitment of GTBP-1 to granules and vice versa. However, TIAR-1 granule formation is not affected by loss of either PQN-59 or GTBP-1, and is only slightly reduced upon loss of both proteins. Given that PQN-59 and GTBP-1 directly interact, this raises the possibility that PQN-59 might have a specific role in GTBP-1 recruitment to granules, but not total SG formation. Therefore, the authors should consider examining additional SG markers including polyA to test whether PQN-59 is required for bona fide SG assembly, or specific recruitment of GTBP-1.

2. The authors should comment on whether their results are specific to embryos or apply to other tissues.
For example, the reduction in PQN-59 granule formation following gtbp-1 RNAi is quite striking in the germline (Fig S5E). GTBP-1 has also been reported to play an important role in SG formation in C. elegans intestinal cells (Kuo et al 2020).

3. The authors should discuss how their findings in C. elegans relate to previously published reports using other systems. For example, Cirillo et al found that UBAP2L is required for SG nucleation following multiple stresses, yet PQN-59 appears dispensable for TIAR-1 granule formation in C. elegans embryos (Fig 4).
Comparison of these apparent differences might provide novel insight regarding the stress response in different organisms and cell types.

4. The authors perform time courses of GTBP-1 and PQN-59 granule dissolution following removal of heat stress and find that granules dissolve more rapidly in pqn-59 or gtbp-1 mutants (Figs 2C and 3C). However only one representative fixed imaging time course is shown in each figure. The authors should quantify rates of granule dissolution for wt embryos versus the pqn-59 and gtbp-1 mutants. Is the faster dissolution a consequence of a reduction in granule size?

5. The authors use heat shock experiments to explore the role of PQN-59 and GTBP-1 in embryonic viability during stress conditions. Following a 10 min incubation at 34°C, lethality for wt embryos ranged from 70-80%, with no significant difference in the pqn-59 or gtbp-1 mutants (Fig 5F). Given that 70-80% lethality in wt embryos is already quite high, is it possible that differences in the pqn-59 or gtbp-1 mutants might be discernable following milder stress conditions? Have the authors tested differences in viability following other types of stresses?

Minor points

1. In Fig S2B, Abbatemarco et al use a 5 hr treatment with 20 mM Arsenite or a 4 hr treatment with 10 mg/mL Puromycin to induce SG formation. Both are prolonged treatments with quite high concentrations of Arsenite or Puromycin. Have the authors tested whether granules are induced with shorter treatments or lower concentrations of Arsenite or Puromycin? Additionally, the authors comment in Line 135 that “the granules are reversible as they dissolve when stress is removed”. This is true for heat-induced granules, but is not shown for Arsenite or Puromycin induced granules. Have the authors tested whether granules induced by Arsenite or Puromycin are similarly reversible?
2. The RGG domain of UBAP2L is required for normal SG nucleation in cell culture, yet appears dispensable for SG formation in C. elegans (Fig S4). The authors should discuss potential reasons for this difference between homologs. Given that UBAP2L and PQN-59 are ~30% similar, how conserved are the RGG domains between UBAP2L and PQN-59?
3. The authors measure levels of GTBP-1 protein upon PQN-59 depletion (Fig S3A), as well as PQN-59 levels upon GTBP-1 depletion (Fig S5C) and find levels of both proteins to be unchanged. However, in several of the representative images levels of GTBP-1 and PQN-59 appear to be significantly reduced or variable. For example, levels of GTBP-1 in the germline appear much lower following pqn-59 RNAi (Fig S4A) and levels of PQN-59 are quite variable in the gtbp1(ax2029) mutant (Fig 3C). Is this due to differences in staining of fixed embryos in the case of Fig 3C?

© 2021. Published by The Company of Biologists under the terms of the Creative Commons Attribution License (https://creativecommons.org/licenses/by/4.0/).

First revision

Author response to reviewers' comments

We would like to thank the reviewers for their constructive criticisms Here we have addressed in details their comments.

The major changes of this revised version are:

1) We have exposed embryos to arsenite and shown that stress granule formation is inhibited by cycloheximide, additional evidence suggesting that the granules that we observe in the embryo are bona fide stress granules.

2) we have measured PQN-59 and GTBP-1 granule numbers in *tiar-1(tn1543)* mutant embryos as suggested by reviewer 2. This has revealed that granules still form in this mutant but that they are reduced in number and intensity.

3) we have performed FISH with a poly(A) probe in single and double mutants and shown that the number of poly(A) granules does not vary in pqn-59 and gtbp-1 mutants but is reduced in tiar-1 mutants, consistent with the reduction of GTBP-1 and PQN-59 containing granules.

4) we have quantified the dissolution of PQN-59, GTBP-1 and TIAR-1 granules.

5) as suggested by all reviewers, we have moved many figures to the main text and we now provide a table in figure 7I that summarizes the results that we have obtained, which we hope will help the reviewers and the readers to keep track of the findings presented.

All together the new data suggest, as hypothesized by reviewer 3, that GTBP-1 and PQN-59 are important to recruit each other to stress granules but do not regulate the number of stress granules, as detected by poly(A) and TIAR- 1.

Reviewer comments and response

Reviewer 1

The experiments that are presented appear to have been done quite rigorously. For example, antibody staining experiments validate several of the GFP-reporter experiments, and mutations are used to complement RNAi knock- down experiments. Unfortunately, two main problems prevent this work from being of high significance to the field: one, the large number of negative results preclude new mechanistic insights from being revealed; and two, most of the experiments were designed without taking full advantage of the literature, making it difficult to integrate any understanding of PQN-59 and GTBP-1 with what is known about the many RNA-binding proteins that condense into granules in the C. elegans germ line in response to stress. In addition, some of the more interesting aspects of the results are not discussed or included in conclusions. However, even with a revised discussion, I do not think the experiments in this study make a significant contribution to the field of cell biology.

We thank the reviewer for mentioning the rigor with which we have performed the experiments presented in this paper. We respectfully disagree with this reviewer on the statement that this manuscript contains a large number of "negative" results. We show that PQN-59 and GTBP-1 are stress granule components but are not required for stress granule assembly in the C. elegans embryo, a phenotype different from what has been observed in human cells in culture but similar to what has been observed in intestinal stem cells in Drosophila: this is not a negative finding. We agree with reviewer 3 that "this study is significant in providing a second example of an animal model where the "rules" of stress granule assembly from tissue culture do not appear to apply » and in our revised version we made efforts to better convey this message, especially in the discussion (page 13, lines 314-320, page 14, lines 372-378).

Reviewer 1 Comments for the Author:

© 2021. Published by The Company of Biologists under the terms of the Creative Commons Attribution License (https://creativecommons.org/licenses/by/4.0/).

Problems/limitations of the study

1. In Fig. 1, the authors use reporters strains for PQN-59 and GTBP-1 to show that heat stress induces granules in early embryos and in the germ line. The “granules” in the oocytes are more fibrillar than those in the embryo, and appear quite distinct from the large granules of RNA-binding proteins induced by meiotic arrest in oocytes (Jud et al., 2008; Noble et al., 2008; Huelgas-Morales et al, 2016; several other refs). They also seem to differ from the granules induced by heat stress, osmotic stress, and anoxia (Jud et al., 2008; Huelgas-Morales et al, 2016). The authors do not make any comparison between the fibrillar PQN-59 and GTBP-1 heat stress-induced granules, and those described in the literature. One simple question is whether PQN-59 or GTBP-1 co-localize with any of the RNA-binding proteins.

We thank the reviewer for this comment.

It is known from the literature that the stress granule composition/structure differs in different cell-types and after different kinds of stress exposure. In this study we asked whether PQN-59 localizes on stress granules and whether PQN-59 and GTBP-1 play a role in the assembly of stress granules, rather than on the stress granule structure. Our studies focus on the embryo, rather than the germline.
To address the comment of this reviewer, we added a picture that shows the colocalization of GTBP-1 granules with a known stress granule marker and RNA binding protein (TIAR-1) in the germline (Fig. S4A). In our stress conditions, granules do look different from embryos to germlines but we would not consider them “fibrillar”.

2. Some of the description in the text does not seem to match the figures. For example, in Fig. S2B, PQN-59 and GTBP-1 do not seem co-localized in the proximal oocytes at all, and there is no mention of the large GTBP-1- only granules?

We thank the reviewer to draw our attention to this. We have analyzed again all the germlines and we cannot observe GTBP-1 large granules only. Most likely, the two red spots observed in this figure were out of focus structures. To avoid misleading the reader, we have now chosen a different germline. To further address this comment, we also provided the merge inset of the two channels where the colocalization is shown (now new figure 2C).

3. The overall flow of the results is a bit confusing: the questions being asked are about stress responses, yet many figures of data are shown without stress. No explanation or rationale is provided for why those questions or comparisons might be interesting. In fact, it appears that PQN-59 inhibits GTBP-1 granules in no-stress conditions, but promotes GTBP-1 granules during heat stress. There is no discussion of these opposite effects.

We are not sure that we understand this comment. In the original submitted version, figure 1 contained the no Heat Shock (HS) condition as a control and to show the localization of both proteins in embryos and germlines not exposed to stress. A similar control was shown for both proteins in Figure 4 in the tiar-1 depletion. Otherwise, pictures of no HS conditions are in supplementary figures and are shown as controls of the performed experiments. For every experimental procedure involving heat-stress exposure, we include non-heat-exposed samples as control.
It is true that, with the exception of the depletion of PQN-59 which results in GTBP-1 granule formation in the P1 cell even in the absence of stress, in all other conditions and mutants we do not observe differences. It would therefore be possible to remove the controls, if required, but we do prefer to keep them.
To address the comment about GTBP-1 localization in pqn-59 mutants we have now added a sentence in the discussion where we speculate on the possible reasons behind the GTBP-1 granules forming in the pqn-59(RNAi) embryos in absence of stress (page 15, lines 392-394 and 398-403).

4. In several places, comments are made about changes in the size of granules, but no quantitation is presented regarding granule size, and the images do not present obvious changes.

We have attempted to measure granule size but this has turned out not reliable. Because of this,

© 2021. Published by The Company of Biologists under the terms of the Creative Commons Attribution License (https://creativecommons.org/licenses/by/4.0/).

we have removed from the text the mention to the size but we have added quantifications for parameters such as number, intensity and granule dissolution time.

5. In figure 2, there are very interesting differences in the anterior vs. posterior blastomere. This is acknowledged but not probed or discussed. Could the posterior granules be P granules? Do they co-localize with P granule markers?

To address this question, we have crossed the GTBP-1::RFP strain with a strain expressing MEG-3::GFP. MEG-3 is a P granule component that does not dissolve in heat-shock conditions (Lee et al., 2020), contrary to the well-known PGL marker dissolving after heat-stress exposure. The posterior GTBP-1 granules observed after PQN-59 depletion in non-stress condition colocalize with the MEG-3 marker, and they also colocalize with the DCP-1 marker (detecting P bodies) (Fig. S1D-F). This is consistent with P body markers colocalizing with P granules (Gallo et al., 2008)

6. I am not sure I am convinced the granules being studied are stress granules. The authors do not show effects of any stress besides heat stress on embryos; the homology between PQN-59 and UBAP2L is not very strong; PQN- 59 (including its RGG domain) is not required to nucleate the granules – different from UBAP2L; in pqn-59(RNAi) embryos GTBP-1 co-localizes with a P-body marker; the heat stress-induced granules previously described in the literature contain several P-body proteins.

In the previous version we showed that the GTBP-1 and PQN-59 granules form in presence of stress (HS in embryos and HS, arsenite and puromycin in germlines). They dissolve when the stress is released and contain TIAR-1 (another marker of SGs). In addition to this, we now show that they form also following arsenite exposure of embryos, they do not form if embryos are treated with cycloheximide (Fig. 2A, B, text lines 106-114, pages 5-6) and they contain RNAs, as shown by FISH (Fig. 3D, 4C, text lines 148-156, page 7 and 199-203, page 8). All this make us confident that we are indeed looking at stress granules.

7. In Fig. 5, it is not clear why the brood size of wild type is so low?

As described in material and methods the number referred to the brood size in 24 hours interval but this was not clearly stated in the graph. We have now corrected the figure by clearly labelling the Y axis with "number of laid eggs in 24 hours" (Figure 7A,B, text lines, 286-289, page 11). We have also measured the brood size (Figure 7C, lines 291-294, page 11).

8. PQN-59 appears to have an important role in reproduction (Fig. 5). However, the rationale to explore this question was not really provided, and this section of the paper seemed very separate from the focus on stress. It seems straight-forward to probe why PQN-59 affects brood size – are there problems with oocytes, sperm, fertilization, mitosis of the stem cells, increased apoptosis? Similarly, what type of embryonic lethality was observed? Is there an early or synchronous cell cycle arrest? Addressing more of these questions might allow the authors to gain insight into PQN-59 function.

Those are very interesting questions but they are not the focus of this manuscript, which is about the role in stress granule assembly of these proteins. We believe that it is however interesting to inform the field that pqn-59 mutants have defects not related to stress that are not shared by its partner stress granule protein, gtbp-1.

9. The discussion is short and largely repeats the results because there really isn't much to say about the bulk of the data. I do not agree with the interpretation of the effect of depleting PQN-59 and GTBP-1 on TIAR-1 granules. There was no change in the number of granules, yet the authors conclude the two proteins have a role in TIAR-1 granule assembly.

There was a small but significant change in the double pqn-59; gtbp-1 mutant in term of TIAR-1 granule number. However, since the change was small and there was no change in the single mutants, we had discussed this result in a very conservative manner in the submitted manuscript. To address this comment, we have now repeated the experiment and there is still a small reduction in granule number which is however not significant. We have therefore removed the statement in the discussion.

© 2021. Published by The Company of Biologists under the terms of the Creative Commons Attribution License (https://creativecommons.org/licenses/by/4.0/).

10. The organization of the figures is difficult to understand, including the decision to have so many supplemental figures. Some seems to be important to the main story, while others could potentially be omitted. The titles of several suppl. figures do not work because panel A is not at all related to the question in panel B, e.g. SFig.4, SFig.6, SFig.7.

We have now re-organized the figures, moved many figures from the supplementary to the main text and provided a summary table, as suggested by reviewer 3, in Figure 7I. This also resulted in a simplification of the supplementary figures. We hope that the manuscript is now easier to read and that the titles of figures are more appropriate.

Minor comments

1. Some of the writing is imprecise, eg. PQN-59 “fell into” granules.

We have now removed this throughout the text and used “assembled into granules” or “formed granules”

Reviewer 2

Advance Summary and Potential Significance to Field:

Stress granules are organelles that are formed by liquid-liquid phase separation. They have been extensively studied in mammals however in C. elegans there is still so much to learn about how they are formed, what proteins and mRNAs associated with them and their functions. Some of the best know proteins that nucleated stress granules formation in mammals are G3BP1 and G3BP2 and UBAP2L. In this paper Abbatemarco et al. studied GTBP-1 and PQN-59, the homologs of G3BP1 and G3BP2 and UBAP2L, respectively. They showed that these proteins associated to stress granules in embryos and gonads during different stress conditions. Unexpectedly, neither GTBP-1 nor PQN-19 are essential for stress granules formations. Although their absence did have an effect on stress granules appearance. Experiments are well performed and the paper has important contributions to worm stress granules composition by adding two new components, GTBP-1 and PQN-19. It is intriguing that these proteins are not essential for stress granules formation. It is possible that the heat shock was too short and the authors should try to challenge the system with longer stress conditions. It will be important to test if stress granules are still formed if two stress granule components are silenced at the same time. At its present form this paper does not make a significant contribution to understand the function of stress granules.

We thank this reviewer for the constructive comments that allowed us to improve our paper. To answer the comment about the length of the heat-shock, we have always used the condition that was the shortest/lightest but that, at the same time, led reproducibly to stress granule assembly. The reason for this is also that embryos (and worms) do not appreciate the increased temperature (nor drug treatment). We have tried to increase the time of exposure to high temperature and we can still see stress granule formation, in both the wild type and the mutant strains (pqn-59, panel A, and gtbp-1, panel B). We have included an example for the reviewer for the embryo but we have not included these data in the manuscript. We refer to the Carlston paper (preprint) for an example of longer stress exposure of the germline (3 hours, Fig. 5).

© 2021. Published by The Company of Biologists under the terms of the Creative Commons Attribution License (https://creativecommons.org/licenses/by/4.0/).

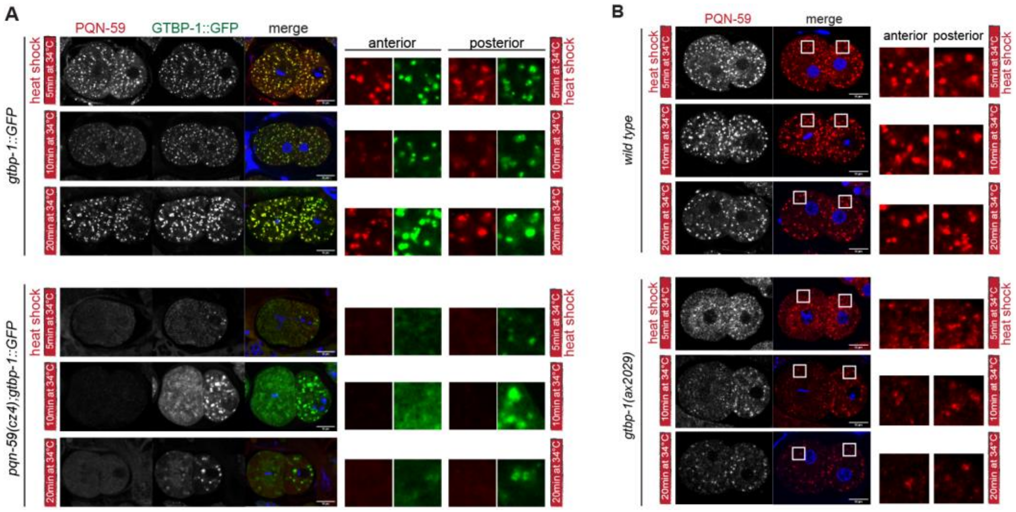

Reviewer 2 Comments for the Author:1. The paper should be reorganized in a way that it is easier for the readers to follow. There are many supplementary figures that should be main figures. It is hard to follow going back and forward from supplementary figures to main figures several times.

We have now moved many figures to the main text and, as suggested by reviewer 3 we provide a table that summarizes the results in Figure 7I.

2. It is intriguing that pqn-59::gfp and gtbp-1::rfp granules are still present in tiar-1(RNAi) embryos. Ii would be interesting to see if the same results are observed in tiar-1 mutant animals.

We stained tiar-1 mutant embryos exposed to heat shock with PQN-59 antibodies and could still observe granules (Fig. S6 of previous version). In this revised version we have extended our analysis. We have now crossed the tiar-1(tn1543) mutant to the strain expressing PQN-59::GFP and GTBP-1::RFP to quantify the number and intensity of PQN-59 and GTBP-1 granules. This has revealed that both are reduced. We have also performed FISH with a poly(A) probe and this shows that the poly(A) signal is also reduced (number and intensity). These new data are now presented in Fig. 6 and described in page 10-11, lines 266-272. All together the data suggest a model in which GTBP-1 and PQN-59 contribute to each other recruitment to stress granules. TIAR-1 is not strictly required to assemble stress granules but, when mutated, less granules containing GTBP-1, PQN-59 and mRNAs are observed.

3. TIAR-1 is essential for stress granules formation in the gonad. Authors should show if in tiar-1 mutant animals GTBP-1 and PQN-19 granules are still present.

In our hands the in the tiar-1 mutant exposed to HS, some granules of GTBP-1 and PQN-59 are still observed in the germline as we show in Figure S5C

Minor points:

-Fig 1D and E. Insets in no heat shock conditions do not have good resolution. They just look like green and red squares.

We have done our best to improve the images. These are pictures taken with an epifluorescence microscope (which we now mention in the figure legend). Unfortunately, we do not have a set up that allows us to heat-shock and image germlines with a better resolution. Most granules dissolve in 5-10 minutes, which leaves very little time. We have taken pictures of the non-heat-shocked germlines in the same conditions. The signal of PQN-59 and GTBP-1 is diffused in the cytoplasm in non-stress condition. Therefore, no structure is detected in the insets, but the diffuse and homogeneous protein distribution can be appreciated.

-Fig 3A. Insets in GTBP-1::RFP do not have good resolution. They just black squares. There is a similar problem in several figures.

© 2021. Published by The Company of Biologists under the terms of the Creative Commons Attribution License (https://creativecommons.org/licenses/by/4.0/).

Some of the insets are the ones which show the efficiency of protein depletion (hence why black). However, we realized that this was sometimes misleading in term of the order of the insets compared to the main image (and Fig. 3A is an example). We have addressed this comment by labelling the insets of each figure to clearly state which protein is shown.

-There is a hole between blastome AB and P1 in the middle of the heat shock embryo in pqn-59(cz4);gtbp- 1::GFP picture. Could authors present a better picture?

We now show a different embryo.

-Page 6, line 146. The depletion of PQN-59….Do authors refer to silencing as depletion? It is confusing.

We have always referred to silencing with pqn-59(RNAi) or PQN-59 depletion. This is common in the cell division/cell polarity C. elegans field. We have attempted to clarify this in the text by writing RNAi depletion of PQN-59 and clarified the nomenclature at the beginning, page 6, lines 133-134).

-Figure S3C. There are more DCP-1 granules in the pqn-59(RNAi) embryo than in the control. Is that correct?

The images that we showed in the original manuscript did suggest a small difference. To address this comment, we have now quantified the number of DCP-1 granules and their intensity and found that the number of DCP-1 granules is not significantly different between control and pqn-59(RNAi) embryos (Fig. S1E). Because of this, we have chosen a more representative picture.

Reviewer 3

Advance Summary and Potential Significance to Field:

In this study Abbatemarco et al use C. elegans embryos and germ cells to investigate the mechanisms controlling dynamics of stress granules (SGs), membraneless RNA/protein assemblies that form in response to various stresses. The authors focus on two conserved proteins that are well-characterized SG nucleators in yeast and human cells: G3BP/GTBP-1 and UBAP2L/PQN-59. The authors find that GTBP-1 and PQN-59 are dispensable for SG formation. Granule morphology and disassembly , however, is altered following depletion/mutation of either protein.This paper is reminiscent of a paper published last year in JCS that found that SGs in Drosophila also do not require canonical SG nucleators for their formation (Buddika et al., 2020). Unlike the Buddika study, the Abbatemarco only reports on three stress granule components and did not address whether recruitment of RNA to SGs is affected in the mutants. Nevertherless, this study is significant in providing a second example of an animal model where the “rules” of stress granule assembly from tissue culture do not appear to apply. The authors also show that neither G3BP nor PQN-59 depleted embryos are hyper sensitized to heat stress, but the significance of this finding is not clear since these embryos still assemble stress granules (albeit with altered morphology).

We thank the reviewer for the positive assessment of our work.

Reviewer 3 Comments for the Author:

While not essential for assembly, depletion of G3BP or PQN-59 does affect SG morphology (I suggest the authors include a table to summarize these findings, as I found them difficult to keep track of). The observations are mostly descriptive so it was difficult to draw any firm conclusions. Further examination of the SG defects in the absence of PQN-59 or G3BP or both may help draw firmer conclusions. I suggest the authors address at least one of the following questions 1) which protein is required to recruit RNA? is PQN-59 primarily responsible for recruiting G3BP to the granules but not essential for other aspects of granule formation? what is the significance of the accelerated dissolution observed in the mutants?

These are all interesting questions. In the revised version, we have performed in situ hybridization with poly(A) in the single pqn-59 (revised figure 3D and 5C) and gtbp-1 (revised figure 4C and 5C) mutants and in the double one (Figure 5C). Consistent with our results that TIAR-1 granules are still observed in the double mutant, we do also observe ploy(A) granules

© 2021. Published by The Company of Biologists under the terms of the Creative Commons Attribution License (https://creativecommons.org/licenses/by/4.0/).

which colocalize with TIAR-1 (Figure 5C). We also performed poly(A) FISH in tiar-1(tn1543) mutants and found that the number of poly(A) granules is reduced. These results indicate that none of the three proteins analysed in this study is responsible, alone, to recruit mRNA molecules inside the granules, neither are pqn-59 and gtbp-1 together. We still ignore if a unique protein responsible for mRNA molecule recruitment inside the SG exists and which one it is.
These data, together with the fact that TIAR-1 granules are not significantly reduced in the single and double mutants and dissolution dynamics are also not affected (Figure 5), suggest that, as this reviewer mentions above, the main role of PQN-59 and GTBP-1 might be to recruit each other inside the granules. Concerning the faster dissolution, we can only speculate that, while neither PQN-59 nor GTBP-1 are essential for the recruitment of each other, the presence of both stabilizes their localization on stress granules
It is difficult to keep track of the findings. As suggested, we now provide a summary table in figure 7I. We hope that this helps to improve the reading.

1. The authors document that loss of PQN-59 causes altered recruitment of GTBP-1 to granules and vice versa. However, TIAR-1 granule formation is not affected by loss of either PQN-59 or GTBP-1, and is only slightly reduced upon loss of both proteins. Given that PQN-59 and GTBP-1 directly interact, this raises the possibility that PQN-59 might have a specific role in GTBP-1 recruitment to granules, but not total SG formation. Therefore, the authors should consider examining additional SG markers including polyA to test whether PQN-59 is required for bona fide SG assembly, or specific recruitment of GTBP-1.

This reviewer is correct, as mentioned in the comment above. We have now performed poly(A) FISH and these results suggest that PQN-59 and GTBP-1 recruit each other on granules (see answer above). This suggest that GTBP-1 and PQN-59 are, in these conditions, mainly important to recruit each other. Consistent with this, we find poly(A) granules that do not colocalize with GTBP-1 or PQN-59 granules, as highlighted by asterisks in the inset of figure 3D and 4C).

2. The authors should comment on whether their results are specific to embryos or apply to other tissues. For example, the reduction in PQN-59 granule formation following gtbp-1 RNAi is quite striking in the germline (Fig S5E). GTBP-1 has also been reported to play an important role in SG formation in C. elegans intestinal cells (Kuo et al 2020).

These are indeed interesting questions. As suggested by the reviewer, we have discussed this point more in the discussion (line 346-356)

3. The authors should discuss how their findings in C. elegans relate to previously published reports using other systems. For example, Cirillo et al found that UBAP2L is required for SG nucleation following multiple stresses, yet PQN-59 appears dispensable for TIAR-1 granule formation in C. elegans embryos (Fig 4). Comparison of these apparent differences might provide novel insight regarding the stress response in different organisms and cell types.

We have now extended our discussion comparing our finding with human cells and the work in Drosophila by Buddika et al., 2020 (e.g. line 314-320, page 13, 345-356, page 14, 376-378, page 15)

4. The authors perform time courses of GTBP-1 and PQN-59 granule dissolution following removal of heat stress and find that granules dissolve more rapidly in pqn-59 or gtbp-1 mutants (Figs 2C and 3C). However, only one representative fixed imaging time course is shown in each figure. The authors should quantify rates of granule dissolution for wt embryos versus the pqn-59 and gtbp-1 mutants. Is the faster dissolution a consequence of a reduction in granule size?

We have now quantified granule dissolution as shown in figure 3E,F and 4D,E. Whether this is a consequence of granule size is difficult to say. First, we did not succeed in measuring granule size in a reliable manner (as mentioned in our answer to point 4 of reviewer 1). Second, the experiment is done on fixed images at different time point so we cannot correlate the size of a specific granule with its dissolution timing. It could be that despite the fact that granules form, the lack of one of the two partners makes the other one more unstable. Consistent with this, dissolution of the TIAR-1 granules in the gtbp-1 mutant does not change, suggesting that the faster dissolution is the result of a less stable interaction of GTBP-1 or PQN-59 with stress

© 2021. Published by The Company of Biologists under the terms of the Creative Commons Attribution License (https://creativecommons.org/licenses/by/4.0/).

granules when the partner is missing rather than a property of the stress granule per se. We have now extended the discussion on this point (lines 339-343, pages 13-14).

5. The authors use heat shock experiments to explore the role of PQN-59 and GTBP-1 in embryonic viability during stress conditions. Following a 10 min incubation at 34°C, lethality for wt embryos ranged from 70-80%, with no significant difference in the pqn-59 or gtbp-1 mutants (Fig 5F). Given that 70-80% lethality in wt embryos is already quite high, is it possible that differences in the pqn-59 or gtbp-1 mutants might be discernable following milder stress conditions? Have the authors tested differences in viability following other types of stresses?

As suggested by the reviewer, we did treat the embryos with a shorter stress exposure (5 minutes at 34° C) and we did not observe major differences between the different strains. We did not include these results in the manuscript but we have added to this report.

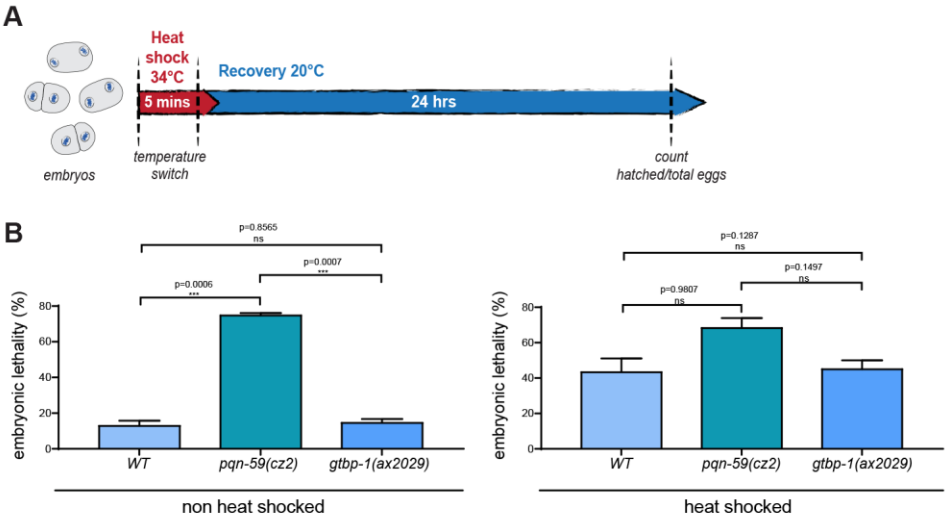

Minor points

1. In Fig S2B, Abbatemarco et al use a 5 hr treatment with 20 mM Arsenite or a 4 hr treatment with 10 mg/mL Puromycin to induce SG formation. Both are prolonged treatments with quite high concentrations of Arsenite or Puromycin. Have the authors tested whether granules are induced with shorter treatments or lower concentrations of Arsenite or Puromycin? Additionally, the authors comment in Line 135 that "the granules are reversible as they dissolve when stress is removed". This is true for heat-induced granules, but is not shown for Arsenite or Puromycin induced granules. Have the authors tested whether granules induced by Arsenite or Puromycin are similarly reversible?

We have tested several conditions with shorter treatments and/or lower concentrations. The conditions showed are the mildest at which we could observe granule formation in a reliable manner. For several drugs, we have used a concentration similar or identical to the one used in previous C. elegans paper (e.g.the puromycin concentration is for example comparable to (Huelgas-Morales et al., 2016, G3), for the cycloheximide, please see (Lee et al., 2020)). In general, because of the egg shell of embryos and the cuticule of worms, worms and embryos do not easily absorb drugs and need to be exposed to 10-30 times higher concentrations than normal (see for example Carvalho et al., 2011, Plos One, Xiong, Pears and Wollard, 2017, Scientific Report, and the website http://www.celescreen.com)

We have attempted to measure reversibility of granules in worms treated with arsenite or puromycin. However, the treatment is harsh and the worms die (as shown in Hunt, PR, Olejnik, N, Sprando, RL, 2012, Food and Chemical Toxicology), preventing us from looking at recovery. As mentioned in our answer to reviewer 1, point 6, we have now exposed embryos to arsenite and showed that arsenite induces formation of granules. Treatment with arsenite and cycloheximide, which inhibits polysome disassembly, prevents stress granule formation, as shown in other systems, again suggesting that GTBP-1 and PQN-59 granules are stress granules.

© 2021. Published by The Company of Biologists under the terms of the Creative Commons Attribution License (https://creativecommons.org/licenses/by/4.0/).

2. The RGG domain of UBAP2L is required for normal SG nucleation in cell culture, yet appears dispensable for SG formation in C. elegans (Fig S4). The authors should discuss potential reasons for this difference between homologs. Given that UBAP2L and PQN-59 are ~30% similar, how conserved are the RGG domains between UBAP2L and PQN-59?

We have now added a sentence in the discussion (lines 345-356, page 14). The RGG is about 18% identical and 30% similar. The fact that it is not required to be recruited to stress granules suggest that this domain is not important for the interaction with GTBP-1 and/or with other stress granule components. We have also added a comparison to the PGL-3 protein which can also assemble into granules when the RGG has been deleted.

3. The authors measure levels of GTBP-1 protein upon PQN-59 depletion (Fig S3A), as well as PQN-59 levels upon GTBP-1 depletion (Fig S5C) and find levels of both proteins to be unchanged. However, in several of the representative images levels of GTBP-1 and PQN-59 appear to be significantly reduced or variable. For example, levels of GTBP-1 in the germline appear much lower following pqn-59 RNAi (Fig S4A) and levels of PQN-59 are quite variable in the gtbp1(ax2029) mutant (Fig 3C). Is this due to differences in staining of fixed embryos in the case of Fig 3C?

The reviewer is right, GTBP-1 levels were lower in the germline shown. To address this comment we have also measured the levels of GTBP-1 in the germlines of pqn-59 depleted worms. In the germlines we find that the levels are reduced (Fig. 3H, text page 7, line 171 and page 8, lines 175-176). We do not think that this is the reason why stress granules are not forming in the oocyte since we still observe granules in the distal germlines. In the embryos (S1B) the levels are very variable but, in average, not significantly different from the control. This is now mentioned in the text, page 7, lines 143-144.

Second decision letter

MS ID#: JOCES/2021/258834

MS TITLE: PQN-59 and GTBP-1 contribute to stress granule formation but are not essential for their assembly in *C. elegans embryos*

AUTHORS: Simona Abbatemarco, Alexandra Bondaz, Francoise Schwager, Jing Wang, Christopher M. Hammell, and Monica Gotta
ARTICLE TYPE: Research Article

We have now reached a decision on the above manuscript.

To see the reviewers' reports and a copy of this decision letter, please go to: https://submit-jcs.biologists.org and click on the 'Manuscripts with Decisions' queue in the Author Area. (Corresponding author only has access to reviews.)

As you will see, the reviewers recognize that many of their major criticisms have been addressed in your revised manuscript. However, they still raised concerns that will require amendments to your manuscript. I hope that you will be able to carry these out, because I would like to be able to accept your paper.

We are aware that you may be experiencing disruption to the normal running of your lab that makes experimental revisions challenging. If it would be helpful, we encourage you to contact us to discuss your revision in greater detail. Please send us a point-by-point response indicating where you are able to address concerns raised (either experimentally or by changes to the text) and where you will not be able to do so within the normal timeframe of a revision. We will then provide further guidance. Please also note that we are happy to extend revision timeframes as necessary.

© 2021. Published by The Company of Biologists under the terms of the Creative Commons Attribution License (https://creativecommons.org/licenses/by/4.0/).

Please ensure that you clearly highlight all changes made in the revised manuscript. Please avoid using 'Tracked changes' in Word files as these are lost in PDF conversion.

I should be grateful if you would also provide a point-by-point response detailing how you have dealt with the points raised by the reviewers in the 'Response to Reviewers' box. Please attend to all of the reviewers' comments. If you do not agree with any of their criticisms or suggestions please explain clearly why this is so.

Reviewer 1

Advance summary and potential significance to field

The authors have made significant revisions to this greatly improved manuscript. I now think that with the minor revisions below, the manuscript is suitable for publication in JOCES. The additional experiments provide a more complete picture of the stress granules described, and how GTBP, PQN, and TIAR-1 interact. Because of the additional experiments, the discussion is much more robust and clarifies the importance of this contribution.

Comments for the author

1. While the organization of the manuscript is much improved, there is some redundancy that could be avoided. In Fig. 1C, the effects of heat stress and recovery are shown for GTBP and PQN. These results are shown again in Figs 3E and 4D. I suggest deleting the repeated images, and just show the effect of gtbp deletion in Figs. 3 and 4, with the graphs showing the comparison.

2. I suggest clarifying the conclusion in line 185 that GTBP granules still form when PQN is absent. This answer varies across the embryo, oocyte, and distal nuclei.

3. Authors should include quantitation of granules for images in Fig. 4F. Are the described dim PQN granules significantly different or not?

4. Authors should include quantitation of granules for images in Fig. S5D. It appears there are fewer PQN granules in the distal nuclei, and maybe oocytes, which is similar to the result in embryos yet it's not quantitated here. Is it also a modest, but significant decrease?

5. While there are now fewer supplemental figures, there are still five. I suggest moving S4B with Fig. 5A, and S4C with Fig. 5C (as is done for 3G and 4F). I was more interested in seeing those no stress controls than others that are included in the main figures.

Reviewer 2

Advance summary and potential significance to field

Stress granules are organelles that are formed by liquid-liquid phase separation. They have been extensively studied in mammals however in C. elegans there is still so much to learn about how they are formed, what proteins and mRNAs associated with them and their functions. Some of the best know proteins that nucleated stress granules formation in mammals are G3BP1 and G3BP2 and UBAP2L. In this paper Abbatemarco et al. studied GTBP-1 and PQN-59, the homologs of G3BP1 and G3BP2 and UBAP2L, respectively. They showed that these proteins associated to worm stress granules in embryos and gonads during different stress conditions. Unexpectedly, neither GTBP-1 nor PQN-19 are essential for stress granules formations. Although their absence did have an effect on stress granules appearance. Experiments are well performed and the paper has important contributions to stress granules composition by adding two new components, GTBP-1 and PQN-19.

© 2021. Published by The Company of Biologists under the terms of the Creative Commons Attribution License (https://creativecommons.org/licenses/by/4.0/).

Comments for the author

The authors have answered all my concerns properly. This manuscript can be accepted for publication

Reviewer 3

Advance summary and potential significance to field

The authors have addressed all my comments. In particular they have done new experiments to demonstrate that 1) the granules they examine are genuine stress granules, 2) stress granules still form in the absence of the canonical stress granule nucleators G3BP and PQN/UBAP2L. Most importantly they now present in situ hybridization experiments against polyA to demonstrate that embryos lacking G3BP and PQN still assemble RNA-rich granules that also contain a third stress granule marker TIA-1.

Together these data demonstrate that findings in tissue culture cells (that claimed that G3BP and UBAP2L are essential for stress granule assembly) do not hold in animals. These findings are in agreement with recent findings in Drosophila intestinal cells where G3BP, TIA and ATX2 were all found to be dispensable for stress granule assembly.

Although these findings may appear superficially as "negative results", they are important because they 1)
challenge the universality of results obtained in tissue culture, and 2)
provide a sound framework on which to base further investigations into G3BP and PQN/UBAP2L functions - abundant proteins whose function remain mysterious.

Comments for the author

The authors may want to comment on TIA-2. Where is it expressed? Could it function redundantly with TIA-1??

Second revision

Author response to reviewers' comments

We thank the reviewers for their positive assessment of our paper. Below we address their remaining minor comments.

Reviewer 1 Advance summary and potential significance to field
The authors have made significant revisions to this greatly improved manuscript. I now think that with the minor revisions below, the manuscript is suitable for publication in JOCES. The additional experiments provide a more complete picture of the stress granules described, and how GTBP, PQN, and TIAR-1 interact. Because of the additional experiments, the discussion is much more robust and clarifies the importance of this contribution.

We thank this reviewer for the positive assessment.

1. While the organization of the manuscript is much improved, there is some redundancy that could be avoided. In Fig. 1C, the effects of heat stress and recovery are shown for GTBP and PQN. These results are shown again in Figs 3E and 4D. I suggest deleting the repeated images, and just show the effect of gtbp deletion in Figs. 3 and 4, with the graphs showing the comparison.

Reply
The experiment in figure 1C is performed in vivo (embryos are heat-shocked under the microscope) and the images are taken with an epifluorescence microscope. In figure 3 and 4, embryos are heat-

© 2021. Published by The Company of Biologists under the terms of the Creative Commons Attribution License (https://creativecommons.org/licenses/by/4.0/).

shocked in an incubator and fixed immediately after the heat-shock. Images are taken with a confocal microscope. We therefore prefer to leave the images of control for each experiment for the readers to be able to compare and not only have the quantifications. It may be repetitive but we do like to include the controls (including the images) for each experiment, whenever possible.

2. I suggest clarifying the conclusion in line 185 that GTBP granules still form when PQN is absent. This answer varies across the embryo, oocyte, and distal nuclei.

Reply
We have now clarified the sentence in line 191-192, page 8

3. Authors should include quantitation of granules for images in Fig. 4F. Are the described dim PQN granules significantly different or not?

Reply
As mentioned in our first revision (minor point of reviewer 2), those are images taken with an epifluorescence microscope. They allow to illustrate the difference in granule formation, but it is not possible to use them for quantification of the granule intensity and number in the same way as for the images taken by confocal microscopy. To address this comment, we have therefore quantified the germlines by using as a readout the standard deviation of the grey value which is high for punctate patterns (like stress granules) and low for more diffuse staining patterns or dimmer granules. Using this method, we have quantified not only 4F (quantifications shown in 4G) and S5D (data shown in S4F and G) but also 3G (quantification in 3I) for completeness. We have added a sentence in material and methods to describe the procedure, line 562-566, page 21.

4. Authors should include quantitation of granules for images in Fig. S5D. It appears there are fewer PQN granules in the distal nuclei, and maybe oocytes, which is similar to the result in embryos yet it's not quantitated here. Is it also a modest, but significant decrease?

Reply
We have quantified these data using the method mentioned above. This does not allow us to quantify the number of granules but results in a difference which is indeed significant only for PQN-59, as the reviewer could rightly observe. We have now added a sentence mentioning this in line 283-284, page 11.

5. While there are now fewer supplemental figures, there are still five. I suggest moving S4B with Fig. 5A, and S4C with Fig. 5C (as is done for 3G and 4F). I was more interested in seeing those no stress controls than others that are included in the main figures.

Reply
We have now moved these panels to the main figure 5. However, to reduce the number of supplementary figures, we also had to move panel S4A together with the previous figure S5, now figure S4. We now have 4 supplementary figures.

Reviewer 2 Advance summary and potential significance to field
Stress granules are organelles that are formed by liquid-liquid phase separation. They have been extensively studied in mammals however in C. elegans there is still so much to learn about how they are formed, what proteins and mRNAs associated with them and their functions. Some of the best know proteins that nucleated stress granules formation in mammals are G3BP1 and G3BP2 and UBAP2L. In this paper Abbatemarco et al. studied GTBP-1 and PQN-59, the homologs of G3BP1 and G3BP2 and UBAP2L, respectively. They showed that these proteins associated to worm stress granules in embryos and gonads during different stress conditions. Unexpectedly, neither GTBP-1 nor PQN-19 are essential for stress granules formations. Although their absence did have an effect on stress granules appearance. Experiments are well performed and the paper has important contributions to stress granules composition by adding two new components, GTBP-1 and PQN-19.

Reviewer 2 Comments for the author
The authors have answered all my concerns properly. This manuscript can be accepted for publication

© 2021. Published by The Company of Biologists under the terms of the Creative Commons Attribution License (https://creativecommons.org/licenses/by/4.0/).

Reply
We thank the reviewer for the very positive assessment.

Reviewer 3 Advance summary and potential significance to field
The authors have addressed all my comments. In particular they have done new experiments to demonstrate that 1) the granules they examine are genuine stress granules, 2) stress granules still form in the absence of the canonical stress granule nucleators G3BP and PQN/UBAP2L. Most importantly they now present in situ hybridization experiments against polyA to demonstrate that embryos lacking G3BP and PQN still assemble RNA-rich granules that also contain a third stress granule marker TIA-1.
Together these data demonstrate that findings in tissue culture cells (that claimed that G3BP and UBAP2L are essential for stress granule assembly) do not hold in animals. These findings are in agreement with recent findings in Drosophila intestinal cells where G3BP, TIA and ATX2 were all found to be dispensable for stress granule assembly.

Although these findings may appear superficially as "negative results", they are important because they 1) challenge the universality of results obtained in tissue culture, and 2) provide a sound framework on which to base further investigations into G3BP and PQN/UBAP2L functions - abundant proteins whose function remain mysterious.

Reply
We thank the reviewer for the very positive assessment.

Reviewer 3 Comments for the author
The authors may want to comment on TIA-2. Where is it expressed? Could it function redundantly with TIA-1??

Reply
This s indeed a very interesting point and we have added a sentence in the discussion, lines 373-377, page 14, saying:
The second C. elegans TIA-1 ortholog, TIAR-2, is expressed in the germline (Jud et al., 2008) and has redundant roles with TIAR-1 in regulating brood size and embryonic viability when the temperature is upshifted from 20 to 25°C (Huelgas-Morales et al., 2016). It will be interesting to investigate whether TIAR-1 and TIAR-2 double depletion results in a stronger reduction in GTBP-1, PQN-59 and poly(A) granule formation when embryos are exposed to stress.

Third decision letter

MS ID#: JOCES/2021/258834

MS TITLE: PQN-59 and GTBP-1 contribute to stress granule formation but are not essential for their assembly in *C. elegans embryos*

AUTHORS: Simona Abbatemarco, Alexandra Bondaz, Francoise Schwager, Jing Wang, Christopher M. Hammell, and Monica Gotta
ARTICLE TYPE: Research Article

I am happy to tell you that your manuscript has been accepted for publication in Journal of Cell Science, pending standard ethics checks.

© 2021. Published by The Company of Biologists under the terms of the Creative Commons Attribution License (https://creativecommons.org/licenses/by/4.0/).
